# Identification of *T. gondii* Myosin Light Chain-1 as a Direct Target of TachypleginA-2, a Small-Molecule Inhibitor of Parasite Motility and Invasion

**DOI:** 10.1371/journal.pone.0098056

**Published:** 2014-06-03

**Authors:** Jacqueline M. Leung, Fanny Tran, Ravindra B. Pathak, Séverine Poupart, Aoife T. Heaslip, Bryan A. Ballif, Nicholas J. Westwood, Gary E. Ward

**Affiliations:** 1 Department of Microbiology and Molecular Genetics, University of Vermont, Burlington, Vermont, United States of America; 2 Program in Cellular and Molecular Biomedical Sciences, University of Vermont, Burlington, Vermont, United States of America; 3 School of Chemistry and Biomedical Sciences Research Complex, University of St Andrews and EaStCHEM, North Haugh, St Andrews, Fife, Scotland, United Kingdom; 4 Department of Biology, University of Vermont, Burlington, Vermont, United States of America; University at Buffalo, United States of America

## Abstract

Motility of the protozoan parasite *Toxoplasma gondii* plays an important role in the parasite’s life cycle and virulence within animal and human hosts. Motility is driven by a myosin motor complex that is highly conserved across the Phylum Apicomplexa. Two key components of this complex are the class XIV unconventional myosin, TgMyoA, and its associated light chain, TgMLC1. We previously showed that treatment of parasites with a small-molecule inhibitor of *T. gondii* invasion and motility, tachypleginA, induces an electrophoretic mobility shift of TgMLC1 that is associated with decreased myosin motor activity. However, the direct target(s) of tachypleginA and the molecular basis of the compound-induced TgMLC1 modification were unknown. We show here by “click” chemistry labelling that TgMLC1 is a direct and covalent target of an alkyne-derivatized analogue of tachypleginA. We also show that this analogue can covalently bind to model thiol substrates. The electrophoretic mobility shift induced by another structural analogue, tachypleginA-2, was associated with the formation of a 225.118 Da adduct on S57 and/or C58, and treatment with deuterated tachypleginA-2 confirmed that the adduct was derived from the compound itself. Recombinant TgMLC1 containing a C58S mutation (but not S57A) was refractory to click labelling and no longer exhibited a mobility shift in response to compound treatment, identifying C58 as the site of compound binding on TgMLC1. Finally, a knock-in parasite line expressing the C58S mutation showed decreased sensitivity to compound treatment in a quantitative 3D motility assay. These data strongly support a model in which tachypleginA and its analogues inhibit the motility of *T. gondii* by binding directly and covalently to C58 of TgMLC1, thereby causing a decrease in the activity of the parasite’s myosin motor.

## Introduction

Parasites of the phylum Apicomplexa are responsible for an enormous amount of morbidity and mortality worldwide; members include *Plasmodium spp*., which cause malaria, and *Toxoplasma gondii*, which infects approximately one-third of the world’s population and can cause life-threatening disease in the developing fetus and immunocompromised individuals. Most apicomplexan parasites need to invade and replicate within cells of their hosts in order to survive. This need can be exploited for drug development, and simultaneously provides an opportunity to investigate the unique biology underlying the apicomplexan life cycle.

A functional myosin motor complex is important for efficient invasion and egress from infected host cells, as well as for parasite-driven dissemination throughout the body [1]. Components of this motor complex include the unconventional class XIV myosin TgMyoA, its associated regulatory light chain, TgMLC1 [2], an essential light chain (TgELC1) [3], and gliding-associated proteins TgGAP40, TgGAP45, TgGAP50 and TgGAP70 [4], [5]. These proteins are localized to the space between the parasite plasma membrane and the flattened vesicles of the inner membrane complex (IMC) [6]–[8]. The motor complex is anchored to the plasma membrane via acylation of TgGAP45 and TgGAP70, and to the IMC through the integral membrane proteins TgGAP40 and TgGAP50 [4], [5]. While many proteins that interact directly and indirectly with TgMyoA have been identified and their physical interactions characterized, how the motor complexes are spatially organized within the parasite and how the components coordinate to produce translational motion are not well understood.

Recent studies have focused on the function of TgMyoA, the protein at the heart of the motor complex. A conditional knockdown approach revealed that *myoA*-depleted parasites were unable to undergo gliding motility, showed severely reduced host cell invasion and egress, and were less virulent in mice [9]. More recently, a system using Cre recombinase-mediated excision generated *myoA* knockout parasites. These parasites were viable but again showed significantly decreased levels of motility, invasion, egress and growth. These findings indicate that while TgMyoA is not strictly essential, it is important for several critical processes in the parasite life cycle [10]. Myosin light chains are typically involved in maintaining the rigidity of myosin motors and regulating actin-activated myosin ATPase activity [11]–[13]. A spectrum of essential and regulatory light chains has been recently discovered in *T. gondii*, with detailed phylogenetic analysis identifying six putative myosin regulatory light chains in addition to TgMLC1 and TgELC1 [14]. Since myosin light chains can modulate the activity of the myosin motor proteins with which they associate, the interfaces between these different light chains and their myosin motors represent promising targets for the design and development of anti-parasitic drugs [15].

We previously performed a high-throughput screen and identified 24 small-molecule inhibitors of host cell invasion by *T. gondii*; 21 of these compounds also inhibited parasite motility [16]. One of the motility inhibitors, tachypleginA, induced a modification on TgMLC1 that increased its electrophoretic mobility [17]. While the modification was mapped to the V46-R59 tryptic peptide of TgMLC1 and found to be associated with a decrease in TgMyoA motor function, the nature of the modification and the direct target(s) of the compound in the parasite were unknown. In this study, we reproduce the compound-induced mobility shift in a heterologous system, and show that the compound binds directly and covalently to C58 of TgMLC1. Knock-in parasites expressing TgMLC1 with a C58S mutation showed significantly reduced sensitivity to compound treatment in a quantitative, Matrigel-based motility assay. These data provide insight into the mechanism by which chemical modification of a regulatory light chain within the class XIV myosin motor complex affects the motility of this important apicomplexan parasite.

## Materials and Methods

### Parasite culture

Parental (RH*Δku80Δhxgprt*) [18], [19], and FLAG-TgMLC1-WT (WT) and FLAG-TgMLC1-C58S (C58S) knock-in *T. gondii* parasites were maintained by serial passage in confluent primary human foreskin fibroblast (HFF) (ATCC CRL-1634) monolayers grown in Dulbecco’s Modified Eagle’s Medium (DMEM), supplemented with 10% (v/v) heat-inactivated fetal bovine serum (FBS) and 10 mM HEPES, pH 7.0, as previously described [20]. The medium was changed to DMEM supplemented with 1% (v/v) heat-inactivated FBS and 10 mM HEPES pH 7.0 just prior to infecting the confluent monolayers with parasites.

### Compound storage and use

All small-molecule inhibitors were synthesized by reacting *N*-*^n^*propyl-4-piperidone with the required aldehyde for 24 h in a solution of acetic acid saturated with dry HCl gas according to previously reported literature protocols [21]–[23] (see Figure S1 and Supporting Information S1 for analytical characterization of novel compounds). Inhibitors were stored at -20°C. Compound stock solutions were prepared by dissolving the solid compound to a concentration of 40 mM in high quality dimethylsulfoxide (DMSO). Immediately before use, compounds were diluted in the appropriate buffer to a final concentration of 100 µM (unless otherwise noted) and incubated in the dark at 25°C for 20 min. A previous report by us showed that double bond isomerisation occurred in a related system in the presence of light [24]. Parasites or baculovirus-infected S*f*9 cells were then incubated in the diluted compound for 20 min at 25°C. Invasion and gliding motility (trail deposition) assays were performed as previously described [16]; 3D motility assays are described further below.

### *Sf*9/baculovirus culture and recombinant TgMLC1 expression and purification

To generate the FLAG-tagged wild-type TgMLC1 baculovirus expression vector (pAcSG2(FLAG-TgMLC1-WT)), the coding sequence for TgMLC1 was amplified by PCR from the *T. gondii* expression vector pTUB-FLAGTgMLC1WT [17] using primers *EcoR*I-FLAG-TgMLC1-Fwd and TgMLC1-*Bgl*II-Rev (see Table S1 for complete list of primers used in this study). The PCR product was cloned in the pGEM T-Easy vector (Promega, Madison, WI), and then subcloned into the pAcSG2 baculovirus expression vector (BD Biosciences, San Jose, CA) using the restriction sites *EcoR*I and *Bgl*II. The FLAG-tagged S57A and C58S TgMLC1 constructs were generated with the QuikChange site-directed mutagenesis method using the primer pairs TgMLC1S57AFwd and TgMLC1S57ARev, and TgMLC1C58S2Fwd and TgMLC1C58S2Rev, respectively, according to the manufacturer’s instructions (Agilent Technologies, Santa Clara, CA). Constructs were verified by diagnostic restriction digests and DNA sequencing.

*Sf*9 cells were infected with recombinant wild-type or mutant TgMLC1 virus and incubated, shaking, for ∼72 h at 27°C. For click chemistry labelling experiments, 1×10^5^ infected *Sf*9 cells were centrifuged for 4 min at 1,000 x *g*, resuspended in 500 µL Hank’s Buffered Salt Solution containing 10 mM HEPES, pH 7.0 (HH), and 100 µM compound or an equivalent amount of DMSO, and incubated at 25°C for 20 min. Cells were centrifuged at 1,000 x *g* for 4 min, washed three times with phosphate-buffered saline (PBS), and extracted in 30 µL lysis buffer (PBS, pH 7.4, 1% (v/v) NP-40 and 1∶100 protease inhibitor cocktail (Sigma-Aldrich, St. Louis MO)) on ice for 2 h. Lysates were then clarified by centrifugation at 21,000 x *g* for 10 min at 4°C prior to labelling (see below).

For mass spectrometry experiments, 5×10^7^ infected *Sf*9 cells were centrifuged for 4 min at 1,000 x *g* and resuspended in 5 mL of HH. The *Sf*9 suspension was split into two equal aliquots, each of which was added to 25 mL of HH containing either 100 µM compound or an equivalent amount of DMSO, and incubated at 25°C for 20 min. Cells were centrifuged for 4 min at 1,000 x *g*, resuspended in 550 µL of *Sf*9 lysis buffer (10 mM imidazole, pH 7.4, 300 mM NaCl, 2 mM EGTA, pH 8.15, 5 mM MgCl_2_, 7% (w/v) sucrose, 3 mM NaN_3_ and 1∶100 protease inhibitor cocktail), and lysed by sonication. The lysate was clarified by centrifugation at 21,000 x *g* (30 min, 4°C). Before use, FLAG affinity resin (Sigma-Aldrich) was equilibrated in FLAG affinity purification buffer (10 mM imidazole, pH 7.4, 300 mM NaCl, 1 mM EGTA, pH 8.15, 5 mM MgCl_2_ and 1∶100 protease inhibitor cocktail). Protein lysates and 100 µL FLAG affinity resin were gently agitated for 1 h at 4°C. Resins were extensively washed with FLAG wash buffer (10 mM imidazole, pH 7.4, 300 mM NaCl, 1 mM EGTA, pH 8.15, 5 mM MgCl_2_ and 1∶500 protease inhibitor cocktail) to remove any unbound proteins. Recombinant FLAG-TgMLC1 was recovered from the resin using two sequential elutions with 250 µL 0.1 mg/mL FLAG peptide (Sigma-Aldrich) in FLAG wash buffer. Eluates were resolved by SDS-PAGE and stained with Colloidal Coomassie Blue. Bands were excised and subjected to in-gel tryptic digestion as described previously [17].

### Click chemistry labelling with tachypleginA-4/biotin-azide

Click chemistry was performed essentially as described [25], [26]. Briefly, protein samples (30 µL aliquots, diluted 1 in 2 with PBS for a starting volume of 60 µL) were treated with 50 µM biotin-azide (50X stock in DMSO), 1 mM tris(2-carboxyethyl)phosphine (TCEP) (fresh 50X stock in ddH_2_O), 100 µM tris-(benzyltriazolylmethyl)amine (TBTA) ligand (17X stock in DMSO:*t*-butanol 1∶4) and 1 mM CuSO_4_ (50X stock in ddH_2_O). Samples were allowed to rock gently at 25°C for 1 h, and then centrifuged at 21,000 x *g* at 4°C to pellet the precipitated proteins. Protein pellets were then mixed with 1X SDS-PAGE sample buffer containing 1.25% (v/v) β-mercaptoethanol and boiled for 10 min.

### Western blotting

Protein samples were resolved by 12% SDS-PAGE and gels were transferred to PVDF-FL membranes, which were blocked with 5% (w/v) bovine serum albumin (BSA) in PBS for 1–12 h. The monoclonal anti-FLAG antibody (Sigma-Aldrich) was used at a 1∶7,500 dilution and the affinity purified polyclonal rabbit anti-TgACT1 antibody (a generous gift from Dr. David Sibley [27]) was used at a 1∶10,000 dilution. Goat anti-mouse IRDye 680RD and goat anti-rabbit IRDye 800 CW infrared dye-conjugated secondary antibodies were used at a 1∶20,000 dilution; the IRDye 800 CW streptavidin was used at a 1∶10,000 dilution. Blots were scanned using an Odyssey LI-COR CLx-0228 imaging system and processed using Image Studio v.2.1.10 software (LI-COR Biosciences, Lincoln, NE).

### Analysis of recombinant TgMLC1 by mass spectrometry

All mass spectrometry preparations were performed in the VGN Proteomics Facility at the University of Vermont. Dimethyl labelling was adapted from protocols described previously [28], [29]. Briefly, lyophilized tryptic peptides were dissolved in 50 µL of 1 M HEPES-NaOH, pH 7.5, and incubated at 25°C for 10 min with 4 µL each of freshly made 4% (v/v) d_0_-formaldehyde in H_2_O and 600 mM sodium cyanoborohydride (NaCNBH_3_) in 1 M NaOH, or 4% (v/v) d_2_-formaldehyde in H_2_O and 600 mM sodium cyanoborodeuteride (NaCNBD_3_) in 1 M NaOH for “light” and “heavy” labelling, respectively. Samples were incubated for an additional 10 min with a second round of labelling reagents, followed by quenching of the reaction with 50 µL of 10% (v/v) trifluoroacetic acid and incubation at 25°C for 1 h. The light and heavy labelled samples were mixed and desalted using PepClean C_18_ spin columns (Thermo Fisher Scientific, Rockford, IL). Spin columns were prewashed twice with 200 µL Buffer B (99.9% (v/v) acetonitrile, 0.1% (v/v) formic acid) and equilibrated with two washes of 200 µL 0.1% (v/v) formic acid. The sample was passed over the column twice to ensure maximal binding to resin, eluted with two rounds of 30 µL Buffer B and dried in a SpeedVac (Thermo Fisher Scientific, San Jose, CA) at 25°C for 2 h.

Mass measurements were made in an LTQ-Orbitrap hybrid mass spectrometer (Thermo Fisher) with a liquid chromatography interface set up as previously described [17]. Precursor scans (360–1600 *m/z*) were conducted in the Orbitrap at 30,000 resolution followed by ten data-dependent MS/MS scans in the LTQ linear ion trap on the most abundant ions identified in the precursor scan. Dynamic exclusion was enabled with a repeat count of three for a duration of 30 s. The lock mass feature for internal calibration was enabled and set to calibrate on the mass of a polydimethylcyclosiloxane ion ([(Si(CH_3_)_2_O)_5_ + H^+^]^+^, *m/z*  =  371.10120) [30], [31].

MS/MS spectra were manually examined for the presence of characteristic “marker” b- and y-ions (b_5_-ion *m/z* = 564.2, b_7_-ion *m/z* = 692.3; y_4_-ion *m/z* = 687.3, y_6_-ion *m/z* = 903.4) calculated using the MS-Product utility program of ProteinProspector v. 5.10.11 (http://prospector.ucsf.edu/prospector/mshome.htm; accessed 2013 Dec 10), for the initial identification of the tachypleginA-2-induced, modified V46-R59 peptide. To collect targeted, high mass accuracy MS/MS spectra, the fragmentation method described above was revised to perform the MS/MS scans in the Orbitrap instead of the LTQ linear ion trap, with a normalized collision energy of 35, activation Q of 0.25, activation time of 30 s and an isolation *m/z* width of 1.2. One MS/MS scan targeted the *m/z* = 849.35115 peak corresponding to that of the modified peptide for fragmentation. MS^3^ analysis was performed with the precursor scan in the Orbitrap followed by MS/MS and MS^3^ scans in the LTQ linear ion trap, with the MS^3^ scan targeting the *m/z* = 1006.40950 peak corresponding to the y_7_-ion.

SEQUEST analysis of tandem mass spectra was conducted using the TgMLC1 amino acid sequence, requiring no enzyme specificity, allowing a 20 ppm window around the precursor mass and allowing differential mass additions of 15.99491 on methionines for oxidation, 79.96633 on serines, threonines and tyrosines for phosphorylation, and the following on cysteines: 0 for a free sulfhydryl, 57.02146 for carbamidomethylation, 71.03711 for acrylamide adduction and 225.11859 for compound-induced modification. For the dimethyl-labelled datasets, a static increase of 28.0313 was set on N-termini and lysines, in addition to a dynamic modification of 6.03766 on N-termini and lysines for heavy labelled peptides.

### Reaction of tachypleginA-4 with a model thiol

Model thiol substrate studies were performed as previously described [32], [33]. tachypleginA-4 (100 mg) was treated with the cysteine model ethyl-2-mercaptoacetate (2.01 eq.) in dichloromethane (DCM, 5 mL) in the presence of triethylamine (2.01 eq.). Upon completion, the reaction mixture was partitioned between H_2_O (25 mL) and DCM (50 mL) and the two layers were separated. The aqueous layer was further extracted with DCM (2×50 mL) and the combined organic extracts were dried (MgSO_4_), filtered and concentrated *in vacuo* to give a bright yellow oil. Purification by column chromatography (hexanes/ethyl acetate: 9/1 to 1/1) afforded the thiol-adduct of tachypleginA-4 as an inseparable mixture of diastereomers (see Results and Supporting Information S1 for more details).

### Cloning and transfection of TgMLC1 knock-in mutants

To construct the FLAG-TgMLC1-WT knock-in plasmid (pFLAGTgMLC1WTAR2tfwd), RH*Δku80Δhxgprt* tachyzoite genomic DNA was extracted using DNAzol Reagent (Invitrogen, Grand Island, NY) followed by ethanol precipitation, and used as template for amplifying the 5’ flanking region for *TgMLC1* (922 bp) with primers TgMLC15’flank*Kpn*IFwd and KozakATGFLAGTgMLC1Rev. The coding sequence for FLAG-TgMLC1 was amplified by PCR from the *T. gondii* expression vector pTUB-FLAGTgMLC1WT [17] using primers KozakATGFLAGTgMLC1Fwd and TgMLC1DHFR3’UTRRev. The 3’ untranslated region (UTR) of the dihydrofolate reductase (DHFR) gene was amplified from TUBIMC1YFP/sagCAT [34] using primers TgMLC1DHFR3’UTRFwd and DHFR3’UTR*Hind*III. Amplicons were resolved by agarose gel electrophoresis, excised and purified using the QIAquick Gel Extraction Kit (QIAGEN, Valencia, CA) and “stitched” together using the fusion PCR technique as described in [35]. The fused, final product was ligated with pGRA1/*ble* [36] via the restriction sites *Kpn*I and *Hind*III to generate the intermediate plasmid pGRA1/*ble*(TgMLC1minigene). The 3’ flanking region of *TgMLC1* was amplified using genomic DNA as described above with primers TgMLC13’flank*Bam*HIFwd and TgMLC13’flank*Xba*IRev, digested with *Bam*HI to yield a 725 bp DNA fragment and purified by gel extraction. The 3’ flanking sequence was then ligated with the intermediate plasmid pGRA1/*ble*(TgMLC1minigene) via *Bam*HI to generate the plasmid pFLAGTgMLC1WTAR. Finally, the tandem tomato (2t) expression cassette was amplified from pCTR_2T_ [37] using primers GRA1*Kpn*IFwd and SAG3’UTR*Bgl*II*Kpn*IRev and cloned nondirectionally into the plasmid pFLAGTgMLC1WTAR to generate the knock-in plasmid pFLAGTgMLC1WTAR2tfwd. The forward orientation of the 2t expression cassette was verified by diagnostic restriction digests. Primers for amplifying the flanking sequences were designed based on the *T. gondii* myosin light chain 1 sequence (TgGT1_257680) in the *Toxoplasma* Genomics Resource ToxoDB (http://toxodb.org/toxo/; accessed 2013 Dec 10 [38], [39]).

The FLAG-tagged C58S TgMLC1 construct was generated with the QuikChange site-directed mutagenesis method using primers TgMLC1C58S2Fwd and TgMLC1C58S2Rev, according to the manufacturer’s instructions (Agilent Technologies, Santa Clara, CA). Constructs were verified by diagnostic restriction digests and sequencing.

100 µg of each of the allelic replacement plasmids was linearized with *Bgl*II and *Pci*I and used for transfection of 2×10^7^ RHΔ*ku80*Δ*hxgprt* parasites. Parasites were resuspended in Cytomix Buffer (120 mM KCl, 0.15 mM CaCl_2_, 10 mM potassium phosphate, pH 7.6, 25 mM HEPES-KOH, pH 7.6, 2 mM EDTA, 5 mM MgCl_2_) supplemented with 2 mM ATP and 5 mM reduced glutathione and electroporated using a ECM 630 electroporation system (BTX, Holliston, MA). Transfected parasites were used to infect confluent HFF monolayers and subjected to two rounds of selection in the presence of 50 µg/mL and 5 µg/mL phleomycin as previously described [36]. Individual parasite clones were isolated by limiting dilution and FLAG-positive, 2t-negative recombinants identified by immunofluorescence microscopy (see below for details). The desired allelic replacement and integration events at the *TgMLC1* locus were evaluated by diagnostic PCRs on tachyzoite genomic DNA prepared as described above, using primer pairs TgMLC15’flankupstrFwd and GRA1*Bgl*IIRev, and TgMLC1+1587(Exon3Start)Fwd and TgMLC13’flankdownstr+829Rev (P1 and P2, and P3 and P4, respectively, in Figure S5).

### Immunofluorescence microscopy

Confluent HFF monolayers were fixed 24 h post-infection in PBS containing 2% (v/v) formaldehyde (30 min, 25°C), then permeabilized in PBS containing 0.25% (v/v) Triton X-100 (15 min, 25°C). Primary and secondary antibodies were diluted in PBS containing 0.5% (w/v) BSA, passed through a 0.22 µm syringe filter and incubated with the samples for 30 min at 25°C. Monoclonal anti-FLAG (Sigma-Aldrich) was used at a dilution of 1∶500 and polyclonal rabbit anti-TgGAP45 (a generous gift from Dr. Con Beckers [5]) was used at a dilution of 1∶1,000. Secondary antibodies conjugated to either Alexa 488 or 546 (Invitrogen) were diluted 1∶500. Images were adjusted for brightness and contrast and pseudocoloured using Adobe Photoshop CS3.

### Cytotoxicity assays

TachypleginA-2 was assayed for cytotoxic effects with the CellTiter-Glo ATP Luminescent Cell Viability Assay Kit (Promega, Madison, WI) using a previously described protocol [16] with minor modifications. Briefly, parasites were harvested by syringe release of infected HFF monolayers through a 27-gauge needle, filtered through a 3 µm Nuclepore filter (Whatman, Piscataway, NJ), centrifuged at 1,000 x *g* for 4 min, and washed and resuspended at a concentration of 1×10^7^ parasites/mL in HH+1% (v/v) dialyzed FBS (Invitrogen). 10 µL of the parasite suspension was mixed with 15 µL of HH containing 25 µM, 50 µM, 100 µM tachypleginA-2 or an equivalent amount of DMSO and incubated for 30 min at 25°C. Two negative controls were used: a “no parasite” control (10 µL of HH in place of the parasite suspension) and a “heat-killed parasite” control (10 µL parasite suspension that was first incubated at 100°C for 10 min). 25 µL of CellTiter-Go reagent (buffer and substrate mix) was added to the parasite suspension, mixed for 2 min and incubated at 25°C for 10 min, followed by luminescence measurements with a Synergy 2 plate reader (BioTek Instruments, Winooski, VT).

### 3D motility assays

Parasites were harvested by syringe release of infected HFF monolayers through a 27-gauge needle, filtered through a 3 µm Nuclepore filter, centrifuged at 1,000 x *g* for 4 min, and washed and resuspended to a concentration of 1-2×10^8^ parasites/mL in 3D Motility Media (1X Minimum Essential Medium lacking sodium bicarbonate, 1% (v/v) FBS, 10 mM HEPES pH 7.0 and 10 mM GlutaMAX L-alanyl-L-glutamine dipeptide) supplemented with 0.3 mg/mL Hoechst 33342. The parasite suspension was mixed with 3 volumes of 3D Motility Media containing 25 µM, 50 µM, 100 µM tachypleginA-2 or an equivalent amount of DMSO and incubated for 20 min at 25°C in the dark, followed by another 3 volumes of Matrigel (BD Biosciences, San Jose, CA), prechilled on ice. Motility was then imaged, tracked and processed as previously described [40]. Parasites with a total trajectory displacement (simple distance from first to last trackpoint) of greater than 2 µm were considered moving based on analysis of a heat-killed parasite preparation, as previously described [40]. Parasites with a total trajectory displacement of 2 µm or less were considered stationary and excluded from further analysis.

Parameters calculated from motility assays were analysed using two-way ANOVA with Sidak’s multiple comparisons test, with GraphPad Prism v. 6.01 (La Jolla, CA). Where statistically significant, multiplicity adjusted P values for comparisons are indicated with asterisks.

## Results

### TgMLC1 is a direct target of the tachyplegin analogue tachypleginA-4

**Figure 1:**
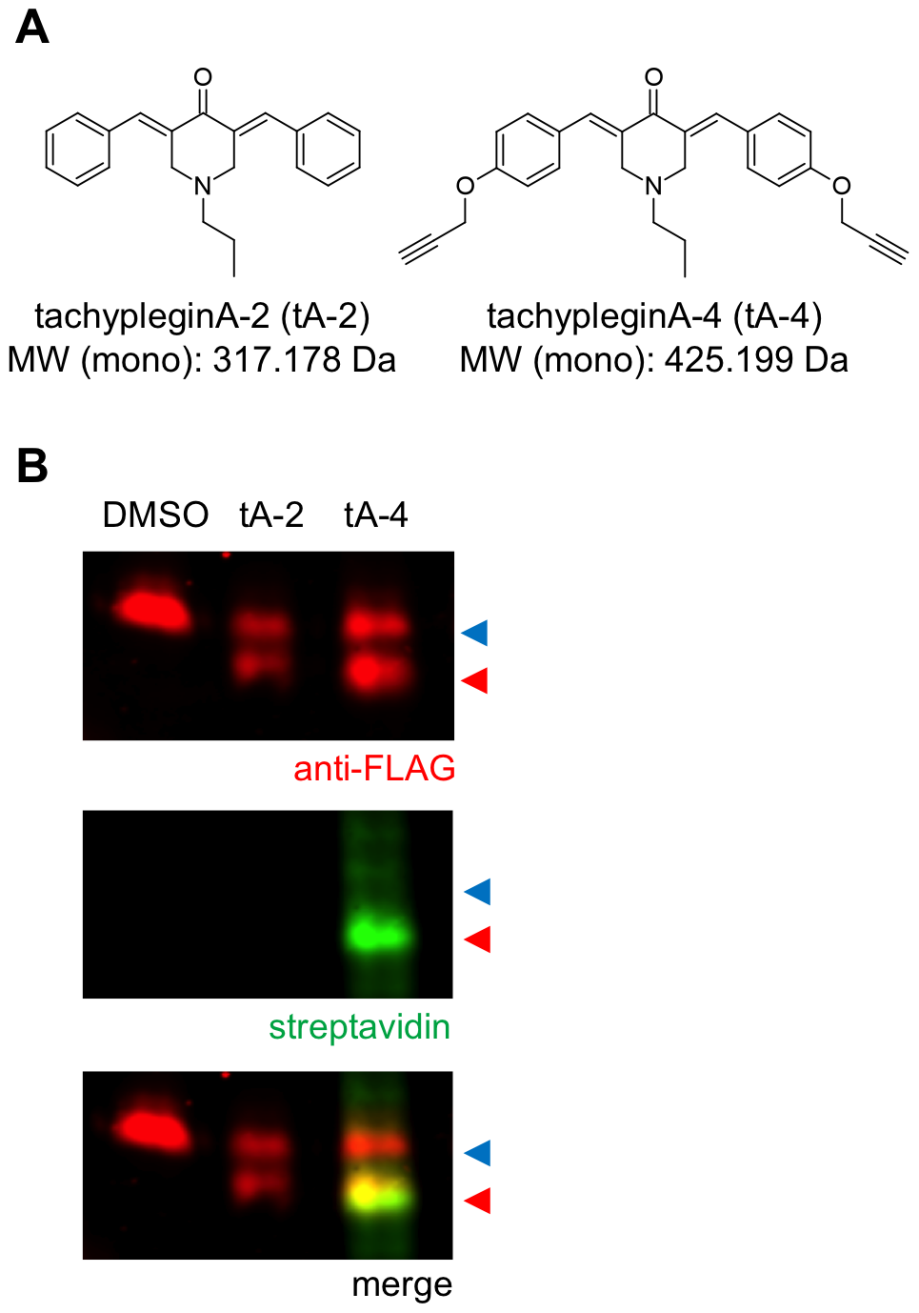
The tachyplegin analogue tachypleginA-4 is covalently bound to the compound-induced, faster-migrating form of rTgMLC1. (**A**) Structures and monoisotopic molecular weights (MW (mono)) of the tachyplegin analogues tachypleginA-2 (tA-2) and the alkyne-containing tachypleginA-4 (tA-4). (**B**) Infected *Sf*9 cells expressing recombinant FLAG-tagged wild-type TgMLC1 (rTgMLC1) were treated for 20 min with 100 µM tA-2, tA-4 or an equivalent amount of DMSO. Cell lysates were then prepared, labelled with biotin-azide and resolved and visualized by SDS-PAGE/western blotting. Both tA-2 and tA-4 induce a shift in the electrophoretic mobility of rTgMLC1 (anti-FLAG western blot, top panel: red arrowhead, modified rTgMLC1; blue arrowhead, unmodified rTgMLC1). However, only the lower form of tA-4-treated rTgMLC1 was labelled by streptavidin (streptavidin western blot, middle panel), indicating that the compound is exclusively bound directly and covalently to the faster-migrating form of rTgMLC1.

As a first step to identifying the target(s) of the tachyplegin family of compounds, we used established methods to prepare an alkyne-derivatized analogue of tachypleginA, tachypleginA-4 (Figures 1A and S1). As previously reported [17] for tachypleginA and tachypleginA-2 (Fig. 1A), treatment of parasites with 100 µM tachypleginA-4 inhibited parasite motility and invasion and resulted in the appearance of a faster-migrating electrophoretic form of TgMLC1 (data not shown). To generate sufficient quantities of the different electrophoretic forms of TgMLC1 for biochemical/proteomic analysis, we tested whether recombinant FLAG-tagged TgMLC1 (rTgMLC1) expressed in insect cells was sensitive to compound treatment. As with native TgMLC1 in parasites, rTgMLC1 underwent a mobility shift in response to treatment of intact S*f*9 cells with either tachypleginA-2 or tachypleginA-4 (Figure 1B).

TachypleginA-4 contains two alkyne functional groups, which were incorporated for target identification purposes. If tachypleginA-4 covalently binds to a protein target, then the alkyne(s) present in the compound could be conjugated to biotin-azide using copper-based “click” chemistry, and subsequent use of a streptavidin probe would enable the detection of biotin and hence protein(s) containing bound tachypleginA-4. Accordingly, we treated rTgMLC1-expressing insect cells with tachypleginA-4, click labelled extracts with biotin-azide and then determined whether biotin was bound to a protein co-migrating with rTgMLC1 by streptavidin western blotting. The major tachypleginA-4/biotin-labelled band from the insect cells co-migrated precisely with the faster-migrating form of rTgMLC1 (Figure 1B). These results indicate that the faster-migrating, compound-induced form of rTgMLC1 contains covalently bound tachypleginA-4 and strongly suggest that the tachyplegin family of compounds binds directly and covalently to TgMLC1.

### TachypleginA-2 binds to and leaves a 225.118 Da adduct on S57 and/or C58

**Figure 2:**
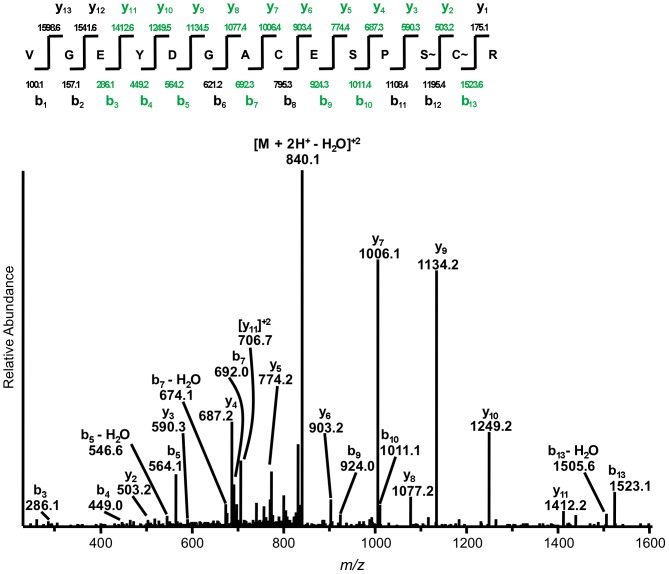
TachypleginA-2 treatment generates an adduct of 225.118 Da on S57 and/or C58 of rTgMLC1. Low energy collision-induced dissociation MS/MS spectrum for the doubly-charged ion corresponding to a modified form of the tryptic V46-R59 peptide. This spectrum was averaged from three independent scans, and is representative of three independent experiments. S∼ and C∼ indicate serine and cysteine residues with a combined adduct mass of 225.118 Da. Coverage of the b- and y-ions in this modified peptide is indicated in green.

We next performed liquid chromatography-tandem mass spectrometry (LC-MS/MS) to map the precise site(s) of compound binding on TgMLC1. Our previous results suggested that the VGEYDGACESPSCR tryptic peptide (V46-R59) contained the site of compound-induced modification [17]. We developed a two-fold strategy to identify peptides in the faster-migrating form of rTgMLC1 whose MS/MS spectra indicated that they could be related to the V46-R59 peptide. If the modification(s) occurred on the N-terminal half of the peptide, then modified fragment ions could be found by manually filtering MS/MS spectra for any characteristic cluster of *m/z* peaks that correspond to the y-ions from the C-terminal half of the unmodified V46-R59 tryptic peptide. Similarly, the MS/MS spectra could be searched for a cluster of *m/z* peaks that correspond to the b-ions from the N-terminal half of the unmodified V46-R59 tryptic peptide, to detect any modification(s) that occurred on the C-terminal half of the peptide. Using this approach, we identified a peptide with *m/z* = 849.351 in the faster-migrating form, whose MS/MS spectra bore close resemblance to that of the unmodified V46-R59 tryptic peptide (Figure 2). The coverage of b- and y-ions was sufficient to determine that, in contrast to the unmodified peptide where both C53 and C58 were alkylated as a result of iodoacetamide treatment [17], C53 was a free sulfhydryl, and importantly, an additional mass of 225.118 Da was observed on C58 and/or S57 (Figure 2). Although we were unable to resolve the exact site(s) of the modification due to the lack of a high confidence b_12_ or y_2_ ion, the mass and location of the adduct(s) to S57 and/or C58 were confirmed on this related peptide using high mass accuracy mass spectrometry and MS^3^ analysis (Figures S2 and S3). Given the expected increased propensity for 1,4-conjugate addition of the soft sulfur atom on C58, compared with the hard oxygen atom on S57 (reviewed in [41]), it seemed reasonable to conclude that C58 was involved in the reaction with compound. Studies on the reaction of tachypleginA-4 with a model thiol were consistent with this assumption (see Figure S4 and Supporting Information S1 for details).

**Figure 3:**
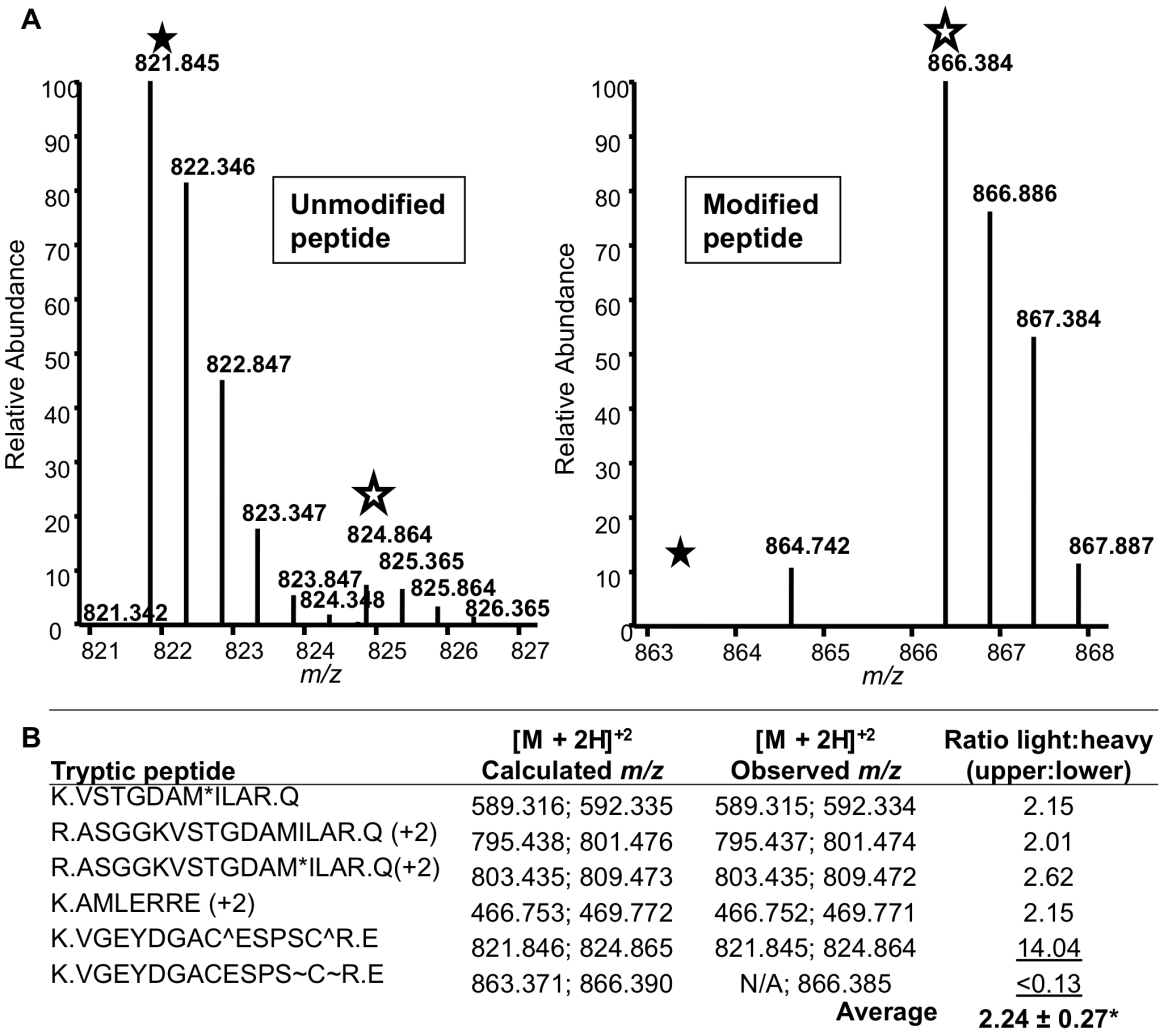
The modified V46-R59 peptide is quantitatively enriched in the faster-migrating, compound-induced form of rTgMLC1. (**A**) The averaged light and heavy isotopic envelopes from the unmodified and modified (faster-migrating) forms of rTgMLC1, respectively, for the unmodified (left panel) and modified (right panel) V46-R59 peptide. Ratios for dimethyl labelled samples were generated by comparing the average relative abundances of the light *vs*. heavy monoisotopic peaks (filled and open stars, respectively). (**B**) The dimethyl labelling ratios (light:heavy) for the four most readily identifiable peptides, in addition to the two forms of the V46-R59 peptide in the unmodified and modified rTgMLC1 forms. The dimethyl labelling ratios for the unmodified and modified V46-R59 peptide (14.04 and < 0.13, respectively, underlined) were strikingly different from the ratio calculated using the other four peptides (2.24±0.27, average±standard deviation). Note that the ratio for the modified peptide was calculated using the abundance of a peptide (*m/z* = 864.742) closest to the expected *m/z* for the light form, since this unmodified form of rTgMLC1 peptide (calculated *m/z* = 863.371) could not be detected. Results are representative of two independent experiments.

To quantify the relative abundance of this related peptide, we performed stable isotope dimethyl labelling on tryptic peptides from the unmodified and modified (*i.e.*, faster-migrating) forms of rTgMLC1 with “light” and “heavy” isotopes, respectively [28], [29]. Since light and heavy labelled peptides with identical amino acid sequences should have closely overlapping chromatographic profiles yet differ in mass, the relative abundance of each peptide in the unmodified and modified forms can be readily determined by calculating its light:heavy ratio, in the same way that relative abundances are calculated with a stable isotope labelling by amino acids in culture (SILAC) approach [42]. Six pairs of tryptic peptides were readily identified from the unmodified and modified forms of rTgMLC1; four of these had similar light:heavy ratios with the light peptide approximately 2.24 fold more abundant than the heavy peptide, suggesting the conversion from the unmodified to the modified form following compound treatment was in this instance less than 50% (Figure 3). The unmodified V46-R59 peptide had a light:heavy ratio of 14.04, reflecting a greater abundance of this peptide in the unmodified form of rTgMLC1 compared to the faster-migrating, modified form (Figure 3A). In contrast, the modified V46-R59 peptide was undetectable in the unmodified form of rTgMLC1 but readily found in the modified form, with an light:heavy ratio of < 0.13 (Figure 3A). These data show that the peptide containing the 225.118 Da adduct is quantitatively enriched in the faster-migrating, compound-induced form of rTgMLC1.

**Figure 4:**
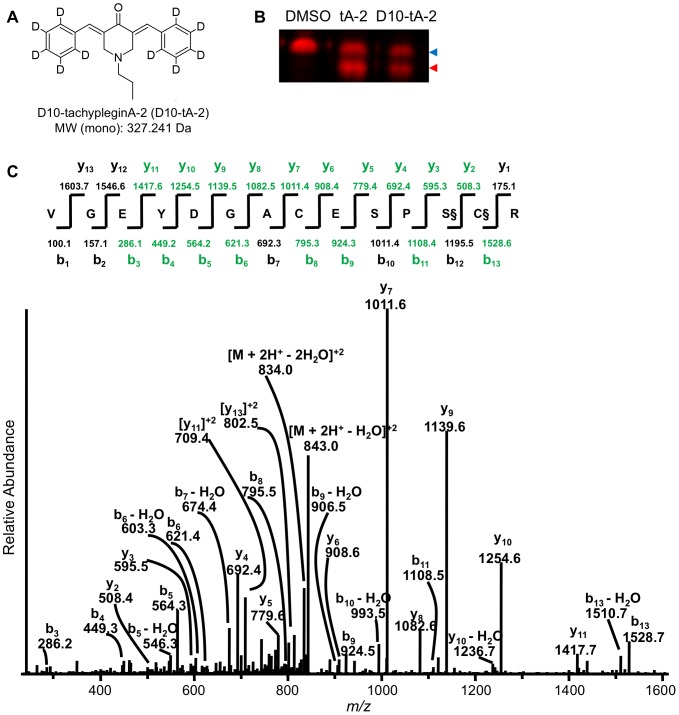
Treatment with the heavy tachyplegin analogue D10-tachypleginA-2 increases the mass of the adduct by 5.03 Da. (**A**) Structure and monoisotopic molecular weight (MW (mono)) of the heavy analogue D10-tachypleginA-2 (D10-tA-2). (**B**) Infected *Sf*9 cells expressing rTgMLC1 were treated for 20 min with 100 µM tA-2, D10-tA-2 or an equivalent amount of DMSO and samples were resolved by SDS-PAGE/western blotting with an anti-FLAG antibody. The unmodified and modified forms of rTgMLC1 are indicated with blue and red arrowheads, respectively. D10-tA-2 induces an electrophoretic mobility shift of rTgMLC1 similar to that observed upon treatment with tA-2. (**C**) Low energy collision-induced dissociation MS/MS spectrum for the doubly-charged ion corresponding to a modified form of the tryptic peptide V46-R59. This spectrum is averaged from twenty independent scans, and is representative of three independent experiments. S§ and C§ indicate serine and cysteine residues with a combined adduct mass of 230.149 Da, which corresponds to an increase in mass of five deuterium atoms. Coverage of the b- and y-ions in this modified peptide is indicated in green.

Given that the mass of tachypleginA-2 is 317.178 Da, the 225.118 Da adduct on S57 and/or C58 could either have been derived from a portion of tachypleginA-2 itself or corresponded to a native posttranslational modification that is induced upon tachypleginA-2 treatment (*e.g.*, upon binding of tachypleginA-2 to some other site(s) on TgMLC1). Searches conducted with the Unimod (http://www.unimod.org/modifications_list.php; accessed 2013 Dec 10), ABRF Delta Mass (http://www.abrf.org/index.cfm/dm.home?AvgMass=all; accessed 2013 Dec 10) and METLIN (http://metlin.scripps.edu/metabo_search_alt2.php; accessed 2013 Dec 10) databases did not return a combination of common posttranslational modifications and/or metabolites that could reasonably account for a total mass of 225.118 Da on serine and/or cysteine residues (data not shown). However, to definitively resolve this question we synthesized a “heavy” tachyplegin analogue, D10-tachypleginA-2 (Figure 4A and S1) that was able to induce the electrophoretic mobility shift (Figure 4B). This analogue contained five deuterium atoms on each of its two phenyl ring substituents for an overall increase in mass of 10.063 Da compared to tachypleginA-2. D10-tachypleginA-2-treated rTgMLC1 samples were subjected to LC-MS/MS with an instrumentation method optimized for fragmentation of *m/z* peaks that corresponded to several possible outcomes: (i) no change in the adduct mass (+225.118 Da); (ii) the addition of five deuterium atoms, if one intact phenyl ring were part of the adduct (+225.118 Da + 5.031 Da  =  +230.149 Da); (iii) the addition of ten deuterium atoms, if both intact phenyl rings were present (+225.118 Da + 10.063 Da  =  +235.181 Da); and (iv) the addition of the full mass of the compound (+327.241 Da). The only modified peptide found in the faster-migrating form of a tachypleginA-2-treated sample was again one with a 225.118 Da adduct (data not shown). This peptide was not detected in the heavy analogue-treated sample. Instead, a new related, modified peptide was present with an isotopic envelope that had increased by 5.031 Da. Annotation of the MS/MS spectra revealed the mass increase (for a new adduct mass of 230.149 Da) localized to S57 and/or C58: in comparison to the spectra shown in Figure 2, all of the detected b-ions remained the same except for the b_13_-ion (whose *m/z* increased by 5.031 Da), and the *m/z* for all of the detected y-ions increased by 5.031 Da (Figure 4C). Taken together, these data demonstrate that the tachyplegin analogues or their metabolites bind directly to rTgMLC1, and this covalent modification results in formation of an adduct on S57 and/or C58 (see Discussion).

### Mutation of C58 prevents the mobility shift and covalent modification of TgMLC1

**Figure 5:**
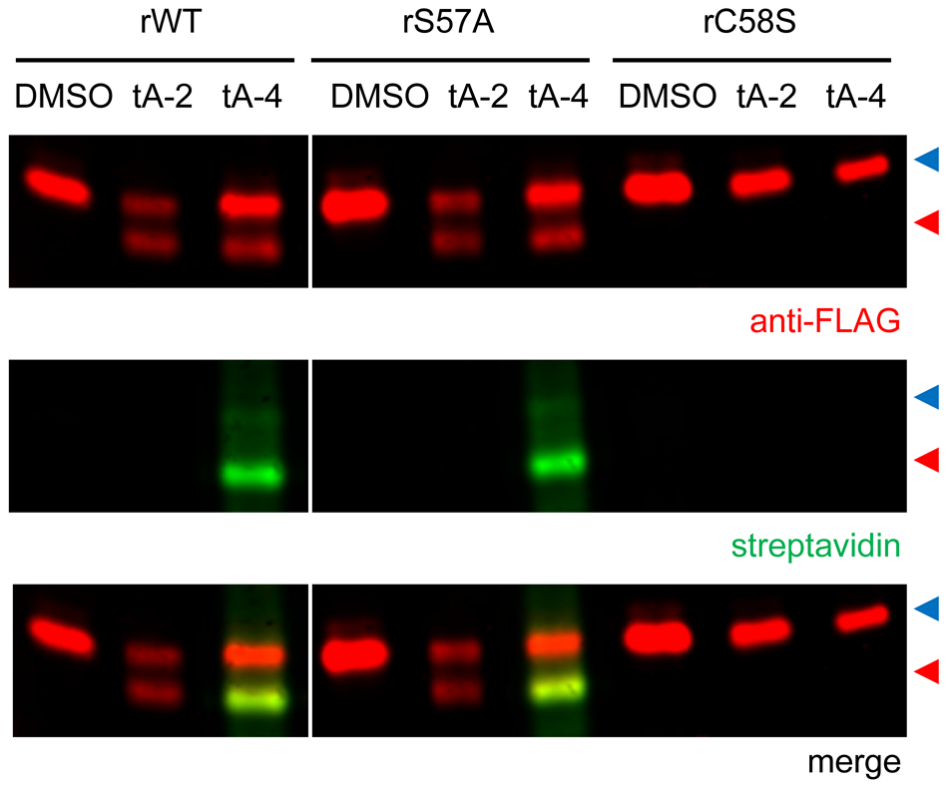
Recombinant TgMLC1 containing a C58S, but not S57A, mutation prevents covalent modification by tachypleginA-4. Infected *Sf*9 cells expressing wild-type rTgMLC1 (rWT), or rTgMLC1 containing either a S57A (rS57A) or C58S (rC58S) mutation were treated for 20 min with 100 µM tA-2, tA-4 or an equivalent amount of DMSO. Cell lysates were then prepared, labelled with biotin-azide (see text for details) and resolved and visualized by SDS-PAGE/western blotting. The unmodified and modified forms are indicated with blue and red arrowheads, respectively. Treatment of rS57A with tA-2 or tA-4 resulted in a TgMLC1 electrophoretic mobility shift and labelling of the faster-migrating of TgMLC1 similar to that observed for rWT, as shown in the anti-FLAG and streptavidin blots, respectively. However, not only did rC58S not shift in response to tA-4 treatment, but no streptavidin signal was detected, indicating that tA-4 was not covalently bound to rC58S. The lanes shown were from the same blot, and were exposed and adjusted for brightness and contrast identically. Results shown are representative of three independent experiments.

To genetically dissect the roles that S57 and C58 play in compound binding, we assessed the ability of tachypleginA-2 and tachypleginA-4 to covalently modify rTgMLC1 containing a mutation at either of these two sites. Like wild-type, rTgMLC1 containing a S57A mutation was able to undergo a mobility shift in response to either tachypleginA-2 or tachypleginA-4 treatment, and a streptavidin signal was detected co-migrating with the faster-migrating, tachypleginA-4-induced form of rTgMLC1 (Figure 5). S57 is therefore neither required for nor the site of compound binding to rTgMLC1. In contrast, the electrophoretic mobility of rTgMLC1 with a C58S mutation did not shift in response to treatment with either of the tachyplegin analogues, and was refractory to labelling and binding by tachypleginA-4 as shown by the lack of a co-migrating streptavidin signal (Figure 5). These results strongly suggest that the compound binds directly to C58 and demonstrate that when this site is mutated to serine, the compound can no longer bind covalently to rTgMLC1 or induce a mobility shift.

### Parasites expressing TgMLC1 with the C58S mutation are less sensitive to tachypleginA-2 treatment in a motility assay

To determine whether the C58S mutation could render parasites resistant to compound treatment, we generated knock-in parasites whose sole endogenous *TgMLC1* allele was replaced with a FLAG-tagged wild-type (WT) or mutant (C58S) *TgMLC1* minigene (Figure S5A). Integration at the desired locus was confirmed by diagnostic PCR on tachyzoite genomic DNA (Figure S5B). These parasites were viable, and immunofluorescence analysis of stable clones showed that both WT and C58S FLAG-tagged TgMLC1 localize to the parasite periphery (Figure S5C). As expected, WT TgMLC1 underwent an electrophoretic mobility shift in response to treatment of the knock-in parasites with tachypleginA-2, whereas C58S TgMLC1 did not (Figure S5D).

**Figure 6:**
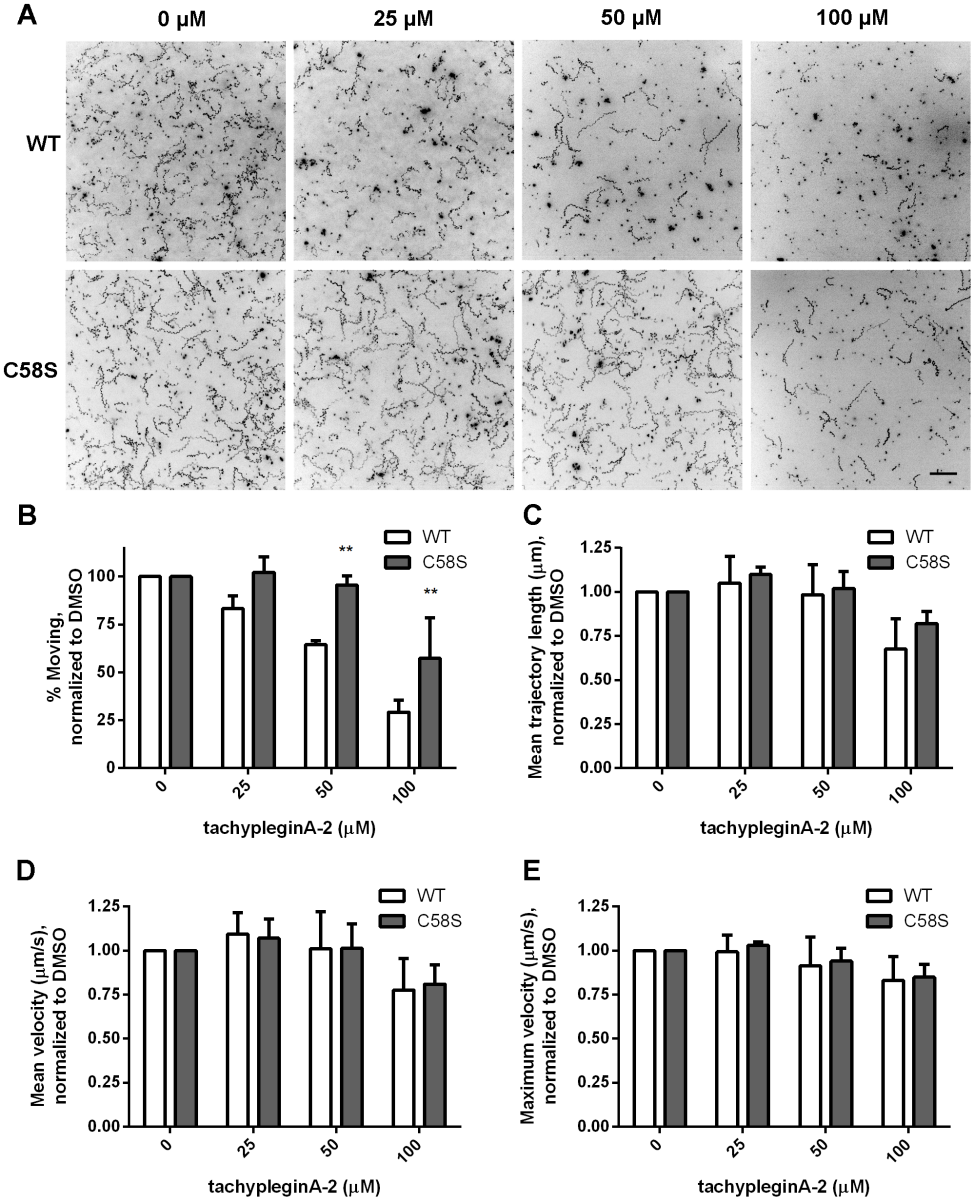
Parasites expressing TgMLC1 with the C58S mutation are significantly less sensitive to the motility-inhibiting effect of tachypleginA-2. (**A**) Maximum intensity projections (MIPs) for FLAG-TgMLC1-WT (WT) and FLAG-TgMLC1-C58S (C58S) knock-in parasites in a 3D motility assay, treated with the indicated concentrations of tA-2. Scale bar = 50 µm. The signal intensities in the MIPs were inverted for better visualization of parasite trajectories. (**B-E**) Graphs comparing the (**B**) percent moving, (**C**) mean trajectory length, (**D**) mean velocity and (**E**) maximum velocity of WT (white bars) and C58S (grey bars) knock-in parasites in the 3D motility assay. All values from compound-treated samples were normalized to those for DMSO; see Figure S6 for the non-normalized data. The total number of WT parasites analyzed was 7,123 for DMSO; 4,662 for 25 µM tA-2; 5,255 for 50 µM tA-2 and 4,328 for 100 µM tA-2. The total number of C58S parasites analyzed was 5,484 for DMSO; 3,325 for 25 µM tA-2; 4,587 for 50 µM tA-2 and 4,417 for 100 µM tA-2. Data shown are the results of three independent experiments, with each experiment performed in triplicate. Datasets were compared by two-way ANOVA (** p<0.001); error bars = standard deviation.

The WT and C58S knock-in parasites were then treated with tachypleginA-2 and assayed for their ability to move in a recently developed 3D Matrigel-based motility assay ([40]; Figure 6A). When the motility parameters were quantified and normalized to those of untreated parasites, treatment of WT parasites with increasing concentrations of tachypleginA-2 was seen to cause a progressive reduction in the percentage of parasites moving (Figure 6B; summarized in Table 1), with no detectable effect on the mean trajectory length, mean velocity or maximum velocity of the parasites that were moving (Figure 6C-E; Table 1). The C58S mutation did not affect basal levels of parasite motility in the absence of compound (see Figure S6 for graphs with non-normalized motility parameters). However, nearly 1.5 times and twice as many C58S parasites remained motile in the presence of 50 and 100 µM tachypleginA-2, respectively, relative to WT parasites. Cytotoxicity assays confirmed that the decrease in percent moving and differences in sensitivity to compound were not due to differential toxicity of the compound in the WT and C58S parasites (data not shown). Although the C58S mutation did not confer complete insensitivity to tachypleginA-2 treatment, these data demonstrate that C58 of TgMLC1 is a physiologically relevant binding site for the small-molecule inhibitor tachypleginA-2.

**Table 1:** Summary of 3D motility parameters for TgMLC1 knock-in parasite lines.

	WT				C58S			
concentration tA-2 used	0 µM	25 µM	50 µM	100 µM	0 µM	25 µM	50 µM	100 µM
n total trajectories analyzed	7,123	4,662	5,255	4,328	5,484	3,325	4,587	4,417
% moving^a^	58.0±12.3%^d^	48.2±10.0%	37.2±6.7%	17.2±5.8%	58.2±6.2%^d^	59.3±4.7%	55.4±3.1%	32.5±9.4%
% fittable moving parasites^b^	83.3±1.1%	75.4±7.3%	76.2±6.6%	75.9±5.1%	81.0±2.5%	84.0±0.8%	82.0±1.3%	83.5±3.6%
mean trajectory length (µm)^c^	26.1±1.7	27.4±4.5	25.5±3.0	17.5±3.6	29.5±2.9	32.5±3.2	30.0±2.1	24.1±0.6
mean velocity (µm/s)^c^	0.8±0.1	0.8±0.1	0.7±0.1	0.5±0.1	0.9±0.2	0.9±0.1	0.9±0.1	0.7±0.1
max velocity (µm/s)^c^	2.1±0.1	2.1±0.1	1.9±0.3	1.8±0.2	2.3±0.2	2.4±0.2	2.2±0.1	1.9±0.0

a Percentage of parasites whose trajectories have a total displacement of > 2 µm.

b Percentage of parasites with a sufficient number of trackpoints (≥ 12) to apply a modified Fourier fit.

c Calculated for fittable trajectories from moving parasites only.

d Values expressed are mean ± SD.

## Discussion

TachypleginA and its analogues were recently identified as inhibitors of *T. gondii* invasion and motility [16], [17], and we show here that TgMLC1 is one of the biologically relevant targets of these compounds using a combination of “click” chemistry, mass spectrometry and mutational analysis. TachypleginA-2 modifies TgMLC1 on C58, and a C58S mutation in TgMLC1 reduces parasite sensitivity to tachypleginA-2 treatment in the 3D motility assay.

While these experiments identify TgMLC1 as a physiologically relevant target of the tachyplegin family of compounds, there are likely to be additional protein targets in *T. gondii*. Experiments in which tachypleginA-4/biotin-azide-labelled samples were resolved by two dimensional electrophoresis revealed streptavidin signals other than the one co-migrating with TgMLC1 (data not shown), and tachypleginA has also been shown to inhibit microneme secretion [16], a process that is not dependent on a functional myosin motor complex [9]. Given the role that certain micronemal proteins play in motility [43], binding of the compound to other targets that act either directly in or upstream of signalling pathways involved in microneme secretion could explain why C58S knock-in parasites still display some sensitivity to treatment with the highest concentration (100 µM) of compound.

Dienones such as the tachyplegin analogues are known to have an affinity for biological thiols, including cysteines [22], and studies performed with EF24, a curcumin (diferuloylmethane) analogue structurally similar to tachypleginA-2, suggested that it could serve as a Michael acceptor and react with thiol-containing molecules such as glutathione and thioredoxin 1 [44], [45]. This reactivity is expected to decrease significantly when the thiol group is replaced with a hydroxyl group [46]–[49], consistent with both our model thiol studies (Figure S4) and our observation that the C58S mutation (but not S57A) disrupts the binding of rTgMLC1 by tachypleginA-4.

Unexpectedly, an adduct of 225.118 Da rather than 317.178 Da (*i.e.*, the intact mass of tachypleginA-2) was observed on C58. One possible explanation for the generation of an adduct of this size is shown in Figure S7. This proposal is consistent with the observation that treatment with D10-tachypleginA-2 resulted in a 5.031 Da increase in the adduct mass (*i.e.*, corresponding to five deuterium atoms), as only one of the two aromatic rings is present in the final adduct. This covalent modification of TgMLC1 by compound appears to only occur when compound is added to intact cells and not to cell lysates. This was true both for TgMLC1 in parasites [17] and rTgMLC1 expressed in *Sf*9 cells (Fig. 1B and data not shown). Perhaps some additional factor present in both *T. gondii* and *Sf*9 cells is needed for binding of the compound, or some condition required for the reaction shown in Figure S7 is met in the cytosol but not in cell extracts. Detailed analytical studies with model peptides, different tachyplegin analogues and a variety of reaction conditions will ultimately be required to elucidate the precise structure of the tachyplegin-derived adduct on TgMLC1 and the mechanism by which it forms in intact cells.

TachypleginA was previously shown to decrease the duty ratio of the *T. gondii* myosin motor complex by 50% in *in vitro* motility assays [17], and we showed here that the compound causes a dose-dependent decrease in the percentage of parasites moving in a Matrigel-based motility assay. Nevertheless, the parasites that moved did so with parameters indistinguishable from those of untreated parasites. In other words, compound treatment significantly decreases the percentage of moving parasites without altering the other motility parameters that can be measured in the 3D assay. This phenotype is largely reversed in the C58S parasites. How binding of tachypleginA-2 to C58 might affect myosin motor function is unclear. C58 is found in the N-terminal extension of TgMLC1, a unique region upstream of the relatively more conserved EF-hand motifs that are a hallmark of calmodulin-like proteins. Although the crystal structure of the C-terminal fragment of *P. yoelii* MyoA in complex with the C-terminal half of *P. falciparum* myosin light chain (PfMTIP) has been solved [50], it lacks the N-terminal extension, making it difficult to predict from these data how covalent modification of C58 of TgMLC1 could affect the interaction of this protein with other components of the myosin motor complex. One intriguing observation is that this cysteine is in close proximity to S55 and S57, two known sites of phosphorylation on TgMLC1 [3], [51]. Future investigation of whether tachyplegin-associated modification of C58 affects the phosphorylation state of these nearby serine residues may shed light on the mechanisms underlying the regulation and function of this class XIV myosin motor complex.

## Supporting Information

Figure S1 **Synthetic scheme for the tachyplegin analogues used in this study: tachypleginA-2, tachypleginA-4 and D10-tachypleginA-2.**
(TIF)

Figure S2 **High mass accuracy mass spectrum of the modified V46-R59 tryptic peptide confirms the site(s) of modification as S57 and/or C58.** Low energy collision-induced dissociation MS/MS spectrum for the doubly-charged ion corresponding to a modified form of the tryptic V46-R59 peptide. Both the precursor (MS) and product (MS/MS) scans were performed in the Orbitrap with the lock mass internal calibration feature enabled for high mass accuracy (*i.e.*, < 3 ppm in these experiments). This spectrum was averaged from three independent scans, and is representative of three independent experiments. S∼ and C∼ indicate serine and cysteine residues with a combined adduct mass of 225.118 Da. Coverage of the b- and y-ions in this modified peptide is indicated in green.
(TIF)

Figure S3 **MS^3^ analysis of the y_7_ ion from the modified V46-R59 tryptic peptide confirms the site(s) of modification as S57 and/or C58.** Low energy collision-induced dissociation MS^3^ (*i.e.*, MS/MS/MS) spectrum for the y_7_ ion derived from the doubly-charged, modified form of the tryptic V46-R59 peptide. The precursor (MS) scan was performed in the Orbitrap and the two product scans (MS^2^ and MS^3^) were performed in the LTQ for maximum sensitivity. This spectrum was averaged from twelve independent scans, and is representative of three independent experiments. S∼ and C∼ indicate serine and cysteine residues with a combined adduct mass of 225.118 Da. Coverage of the b- and y-ions in this modified peptide is indicated in green. Whereas the presence of unexplained fragments is apparent, fragment ions consistent with MS^3^ fragmentation of the MS^2^ y_7_ ion are distinct.
(TIF)

Figure S4 **TachypleginA-4 reacts with thiol-containing compounds.** Previous studies with close structural analogues of the tachyplegin family have shown that this type of compound is susceptible to reaction with thiols, such as those on cysteine residues [32], [33]. To explore this possibility, tachypleginA-4 was reacted with a slight excess of the model thiol-containing compound ethyl-2-mercaptoacetate in the presence of the weak base triethylamine. **(A)** Reaction scheme showing the formation of the diastereomeric mixture of 1,4-conjugated addition derived products **S1** on reaction of tachypleginA-4 with ethyl-2-mercaptoacetate in the presence of triethylamine (Et_3_N) in dichloromethane (DCM). It is important to note that no evidence to support the formation of the alkynylated thiol **S2** was gained in this reaction consistent with the conclusion that TgMLC1 is not labelled by alkynyl transfer from tachypleginA-4 to the protein (as shown in the alternate reaction pathway, data not shown); **(B)** Chemical structure of the six possible diastereoisomers of **S1.** These results demonstrate that tachypleginA-4 can covalently bind to thiols and are consistent with C58 as a feasible site of compound binding. To the best of our knowledge and consistent with the Hard Soft Acid Base (HSAB) theory as reviewed in [41], no examples of the intermolecular 1,4-conjugate addition of an alcohol (such as serine) to compounds like tachypleginA-4 are known.
(TIF)

Figure S5 **Generation and characterization of TgMLC1 knock-in parasite lines. (A)** Schematic depicting the *TgMLC1* locus prior to and after integration of the knock-in DNA fragment by double homologous recombination. White boxes represent regions flanking the *TgMLC1* gene used to target the phleomycin resistance cassette to the *TgMLC1* locus. Dark grey boxes represent predicted exons in the *TgMLC1* locus. Light grey boxes represent elements introduced after double homologous recombination. DHFR  =  dihydrofolate reductase; UTR  =  untranslated region; ble^R^  =  phleomycin resistance cassette; SAG1  =  surface antigen 1. **(B)** PCRs using the primer combinations indicated in (A) and genomic DNA extracted from the clonal parental RH*Δku80Δhxgprt* (*Δku80*), FLAG-tagged wild-type TgMLC1 (WT) or FLAG-tagged C58S TgMLC1 knock-in (C58S) parasites. Expected amplicon sizes for P1 + P2 PCR  =  no product for intact, endogenous *TgMLC1* locus, and 3.5 kb for the *TgMLC1* locus after integration. Expected amplicon sizes for P3 + P4 PCR  =  1.5 kb for intact, endogenous *TgMLC1* locus, and 2.9 kb for the *TgMLC1* locus after integration. Numbers on the left indicate size of DNA fragments in kilobases (kb); L  =  ladder; (-)  =  no template. **(C)** Dual immunofluorescence labelling of knock-in parasites expressing FLAG-TgMLC1-WT (WT) or FLAG-TgMLC1-C58S (C58S) with antibodies against FLAG (green) or TgGAP45 (magenta). Both the wild-type and mutant TgMLC1 localize to the parasite periphery. Note that colocalization of signals from the green and magenta channels produces a white signal in the overlay. Scale bar  =  5 µm. **(D)** WT or C58S parasites were treated with 100 µM tA-2 or an equivalent amount of DMSO, and samples were resolved by SDS-PAGE/western blotting. The unmodified and modified forms of TgMLC1 are indicated by blue and red arrowheads, respectively. Flag-tagged wild-type TgMLC1 was able to undergo an electrophoretic mobility shift in response to the compound whereas the C58S-containing TgMLC1 was not. TgACT1  =  *T. gondii* actin loading control.
(TIF)

Figure S6 **Non-normalized motility parameters of the knock-in parasites upon tachypleginA-2 treatment.** Graphs comparing the **(A)** percent moving, **(B)** mean trajectory length, **(C)** mean velocity and **(D)** maximum velocity of WT (white bars) and C58S (grey bars) knock-in parasites in the 3D motility assay. The total number of WT parasites analyzed was 7,123 for DMSO, 4,662 for 25 µM tA-2, 5,255 for 50 µM tA-2 and 4,328 for 100 µM tA-2; the total number of C58S parasites analyzed was 5,484 for DMSO, 3,325 for 25 µM tA-2, 4,587 for 50 µM tA-2 and 4,417 for 100 µM tA-2. Data shown are the results of three independent experiments, with each experiment performed in triplicate. Datasets were compared by two-way ANOVA (* p < 0.05); error bars  =  standard deviation.
(TIF)

Figure S7 **One possible mechanism to explain the observed formation of tachyplegin-derived adducts on C58 of TgMLC1.** After formation of an initial TgMLC1-tachypleginA-2 adduct (**S3** all Hs) or a TgMLC1-D10-tachypleginA-2 adduct (**S3** all Ds), subsequent nucleophile-induced loss of the second aromatic ring could occur to give **S5** via **S4**. Oxidation of **S5** would then be required to produce the final adducts (the proposed adduct is shown in red for tachypleginA-2 (all Hs) or blue for D10-tachyplegin A-2 (all Ds)). This speculative explanation is consistent with the experimentally observed mass shifts.
(TIF)

Table S1 Primers used in this study.
(DOCX)

Supporting Information S1 (DOC)
