# Supplementary figures and images for: Identification of T. gondii Myosin Light Chain-1 as a Direct Target of TachypleginA-2, a Small-Molecule Inhibitor of Parasite Motility and Invasion
Source: PLoS One. 2014 Jun 3;9(6):e98056. doi: 10.1371/journal.pone.0098056 (PMC4043638; doi:10.1371/journal.pone.0098056)

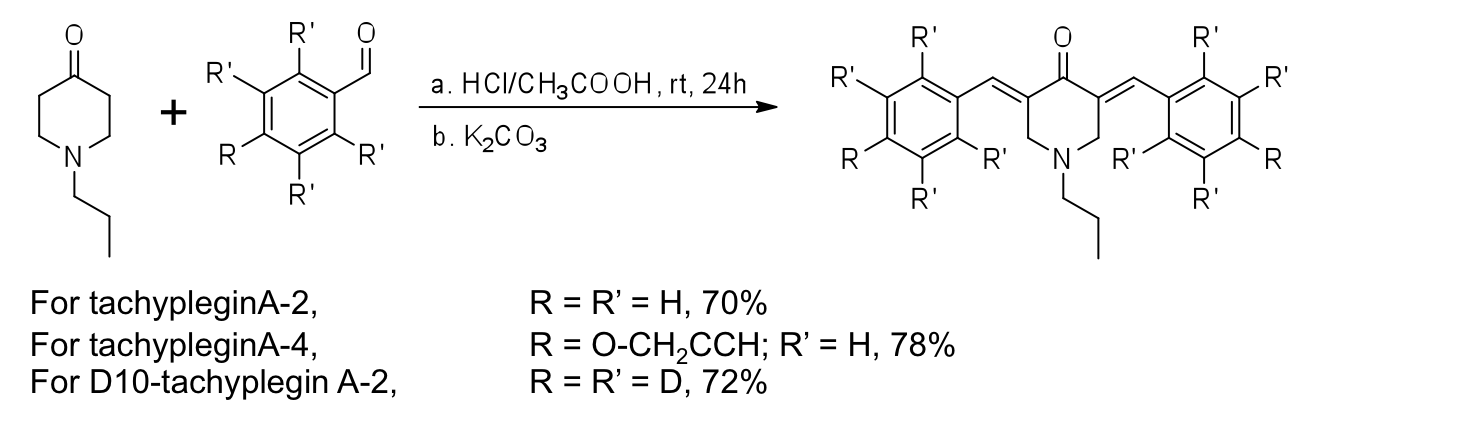

Supplement: Figure S1 — Synthetic scheme for the tachyplegin analogues used in this study: tachypleginA-2, tachypleginA-4 and D10-tachypleginA-2. (TIF) [file pone.0098056.s001.tif]

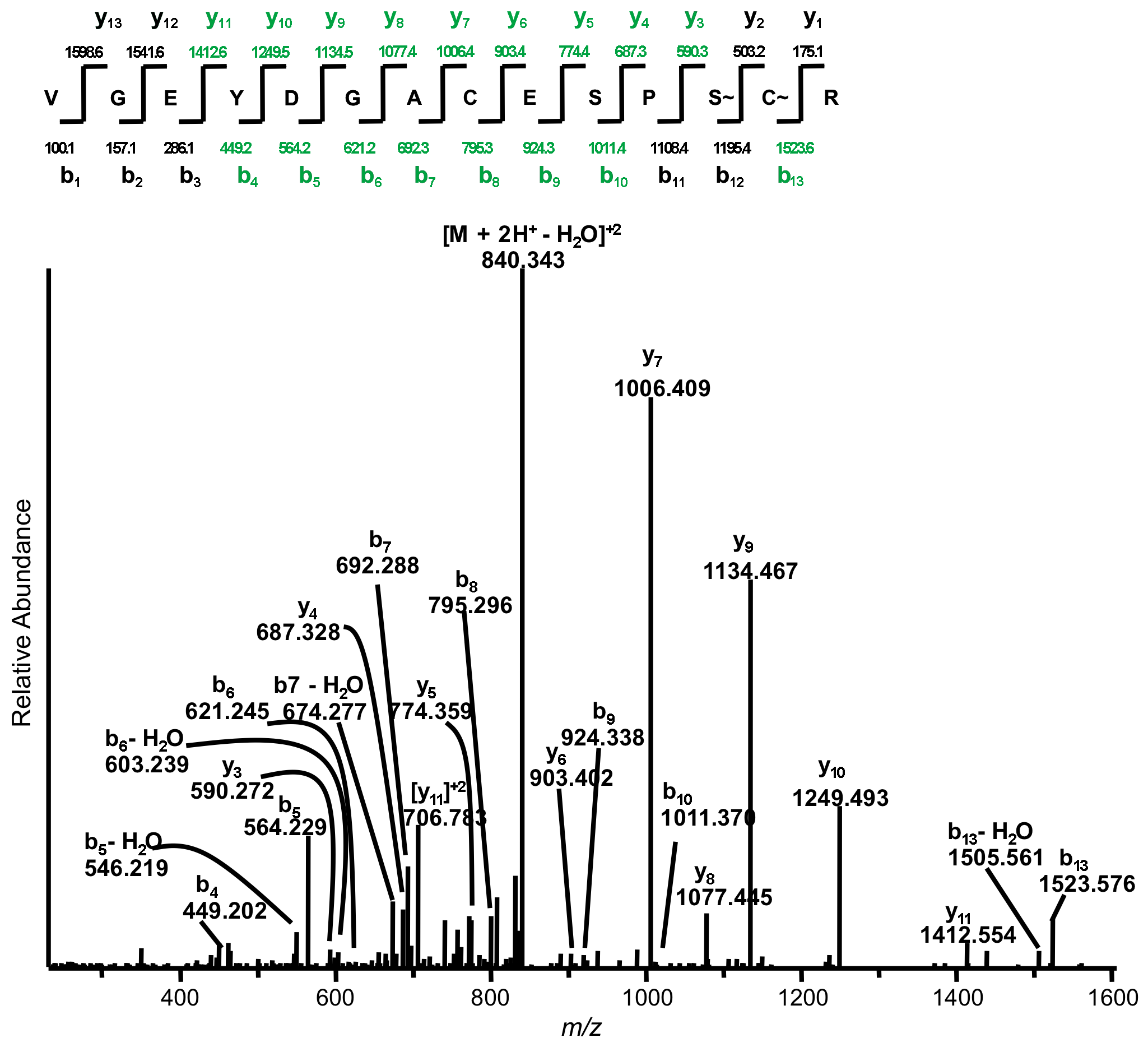

Supplement: Figure S2 — High mass accuracy mass spectrum of the modified V46-R59 tryptic peptide confirms the site(s) of modification as S57 and/or C58. Low energy collision-induced dissociation MS/MS spectrum for the doubly-charged ion corresponding to a modified form of the tryptic V46-R59 peptide. Both the precursor (MS) and product (MS/MS) scans were performed in the Orbitrap with the lock mass internal calibration feature enabled for high mass accuracy (i.e., < 3 ppm in these experiments). This spectrum was averaged from three independent scans, and is representative of three independent experiments. S∼ and C∼ indicate serine and cysteine residues with a combined adduct mass of 225.118 Da. Coverage of the b- and y-ions in this modified peptide is indicated in green. (TIF) [file pone.0098056.s002.tif]

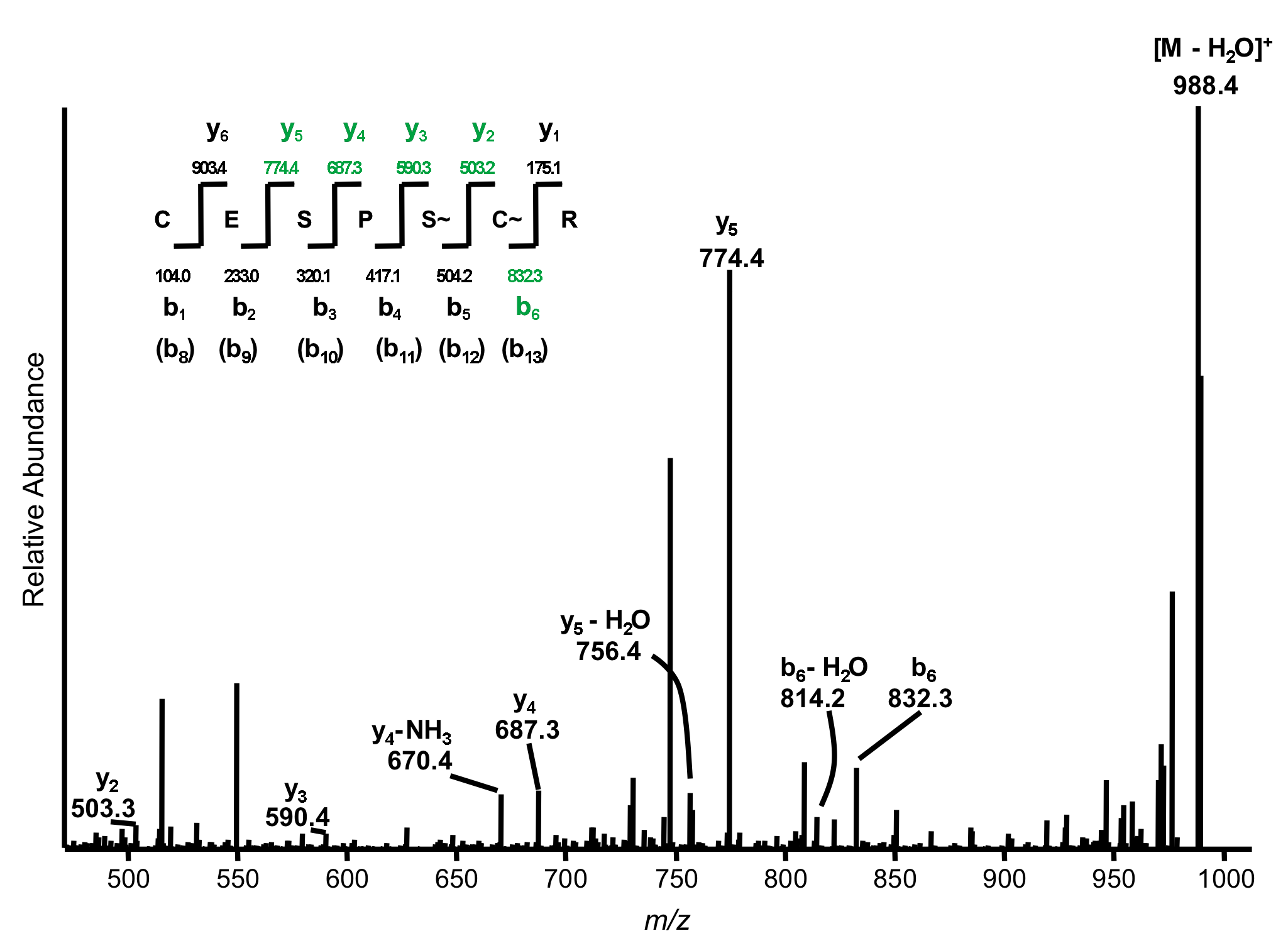

Supplement: Figure S3 — MS3 analysis of the y7 ion from the modified V46-R59 tryptic peptide confirms the site(s) of modification as S57 and/or C58. Low energy collision-induced dissociation MS3 (i.e., MS/MS/MS) spectrum for the y7 ion derived from the doubly-charged, modified form of the tryptic V46-R59 peptide. The precursor (MS) scan was performed in the Orbitrap and the two product scans (MS2 and MS3) were performed in the LTQ for maximum sensitivity. This spectrum was averaged from twelve independent scans, and is representative of three independent experiments. S∼ and C∼ indicate serine and cysteine residues with a combined adduct mass of 225.118 Da. Coverage of the b- and y-ions in this modified peptide is indicated in green. Whereas the presence of unexplained fragments is apparent, fragment ions consistent with MS3 fragmentation of the MS2 y7 ion are distinct. (TIF) [file pone.0098056.s003.tif]

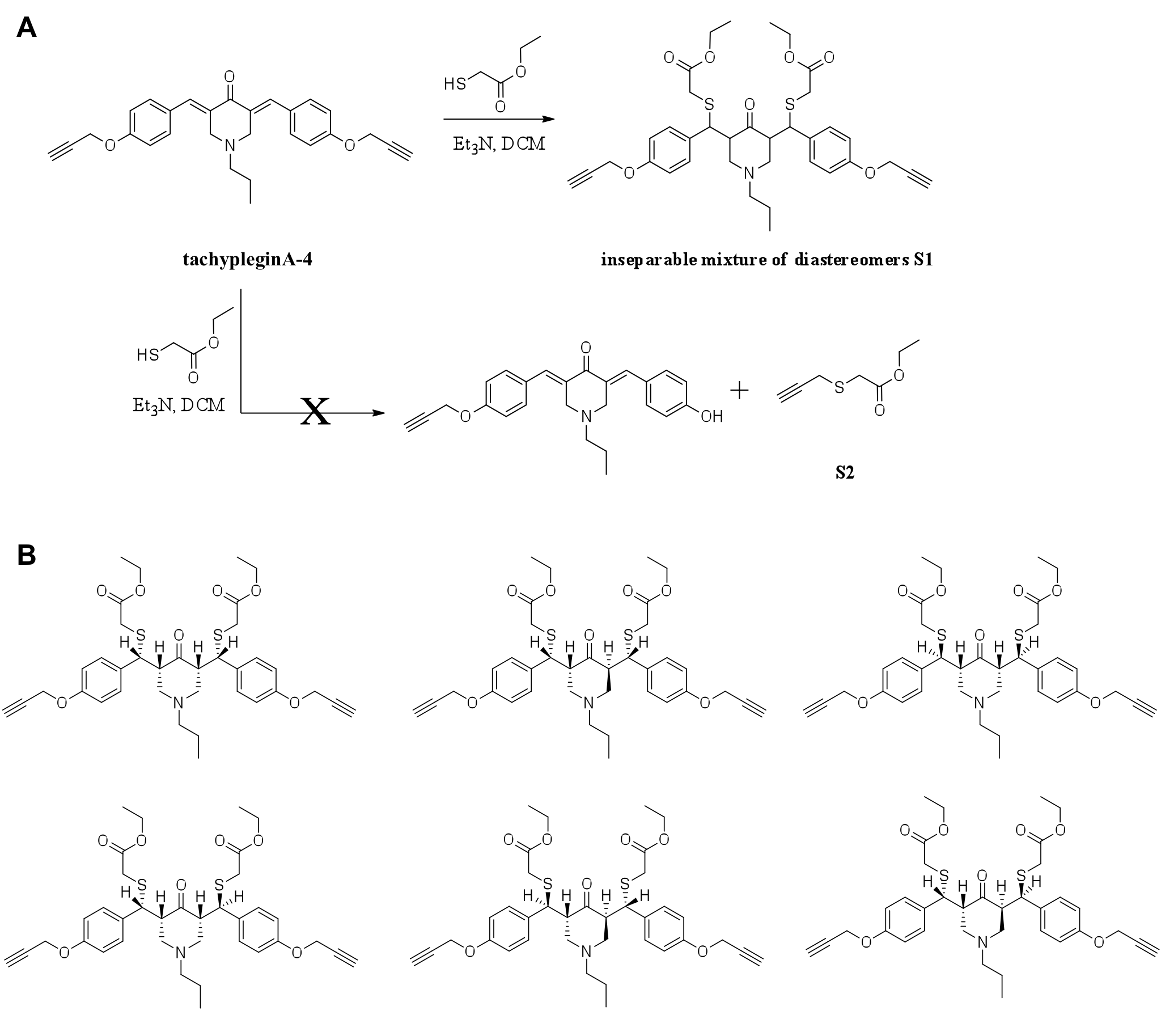

Supplement: Figure S4 — TachypleginA-4 reacts with thiol-containing compounds. Previous studies with close structural analogues of the tachyplegin family have shown that this type of compound is susceptible to reaction with thiols, such as those on cysteine residues [32], [33]. To explore this possibility, tachypleginA-4 was reacted with a slight excess of the model thiol-containing compound ethyl-2-mercaptoacetate in the presence of the weak base triethylamine. (A) Reaction scheme showing the formation of the diastereomeric mixture of 1,4-conjugated addition derived products S1 on reaction of tachypleginA-4 with ethyl-2-mercaptoacetate in the presence of triethylamine (Et3N) in dichloromethane (DCM). It is important to note that no evidence to support the formation of the alkynylated thiol S2 was gained in this reaction consistent with the conclusion that TgMLC1 is not labelled by alkynyl transfer from tachypleginA-4 to the protein (as shown in the alternate reaction pathway, data not shown); (B) Chemical structure of the six possible diastereoisomers of S1. These results demonstrate that tachypleginA-4 can covalently bind to thiols and are consistent with C58 as a feasible site of compound binding. To the best of our knowledge and consistent with the Hard Soft Acid Base (HSAB) theory as reviewed in [41], no examples of the intermolecular 1,4-conjugate addition of an alcohol (such as serine) to compounds like tachypleginA-4 are known. (TIF) [file pone.0098056.s004.tif]

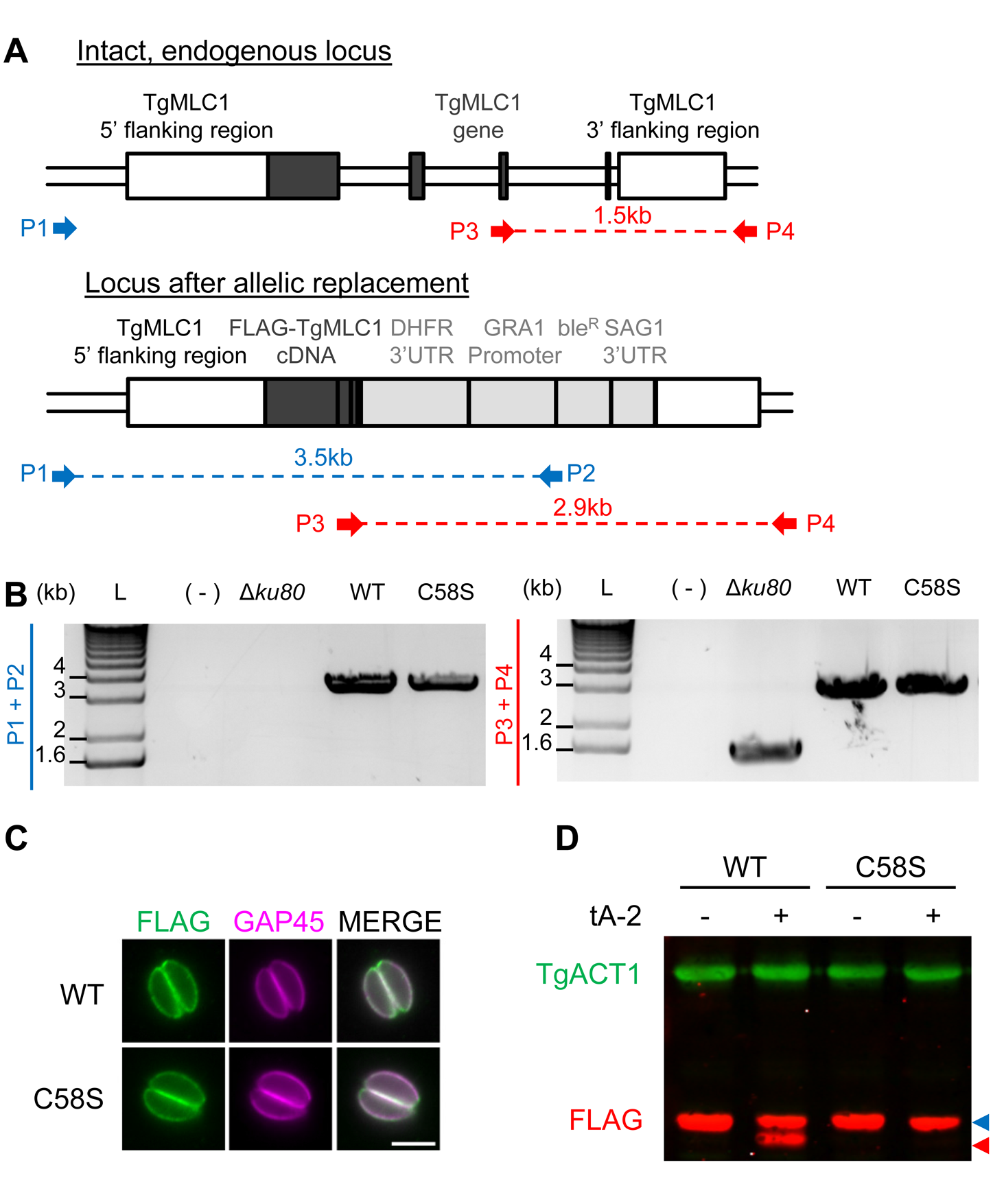

Supplement: Figure S5 — Generation and characterization of TgMLC1 knock-in parasite lines. (A) Schematic depicting the TgMLC1 locus prior to and after integration of the knock-in DNA fragment by double homologous recombination. White boxes represent regions flanking the TgMLC1 gene used to target the phleomycin resistance cassette to the TgMLC1 locus. Dark grey boxes represent predicted exons in the TgMLC1 locus. Light grey boxes represent elements introduced after double homologous recombination. DHFR = dihydrofolate reductase; UTR = untranslated region; bleR = phleomycin resistance cassette; SAG1 = surface antigen 1. (B) PCRs using the primer combinations indicated in (A) and genomic DNA extracted from the clonal parental RHΔku80Δhxgprt (Δku80), FLAG-tagged wild-type TgMLC1 (WT) or FLAG-tagged C58S TgMLC1 knock-in (C58S) parasites. Expected amplicon sizes for P1 + P2 PCR = no product for intact, endogenous TgMLC1 locus, and 3.5 kb for the TgMLC1 locus after integration. Expected amplicon sizes for P3 + P4 PCR = 1.5 kb for intact, endogenous TgMLC1 locus, and 2.9 kb for the TgMLC1 locus after integration. Numbers on the left indicate size of DNA fragments in kilobases (kb); L = ladder; (-) = no template. (C) Dual immunofluorescence labelling of knock-in parasites expressing FLAG-TgMLC1-WT (WT) or FLAG-TgMLC1-C58S (C58S) with antibodies against FLAG (green) or TgGAP45 (magenta). Both the wild-type and mutant TgMLC1 localize to the parasite periphery. Note that colocalization of signals from the green and magenta channels produces a white signal in the overlay. Scale bar = 5 µm. (D) WT or C58S parasites were treated with 100 µM tA-2 or an equivalent amount of DMSO, and samples were resolved by SDS-PAGE/western blotting. The unmodified and modified forms of TgMLC1 are indicated by blue and red arrowheads, respectively. Flag-tagged wild-type TgMLC1 was able to undergo an electrophoretic mobility shift in response to the compound whereas the C58S-containing TgMLC1 was not. TgACT1 = T. gondii a [file pone.0098056.s005.tif]

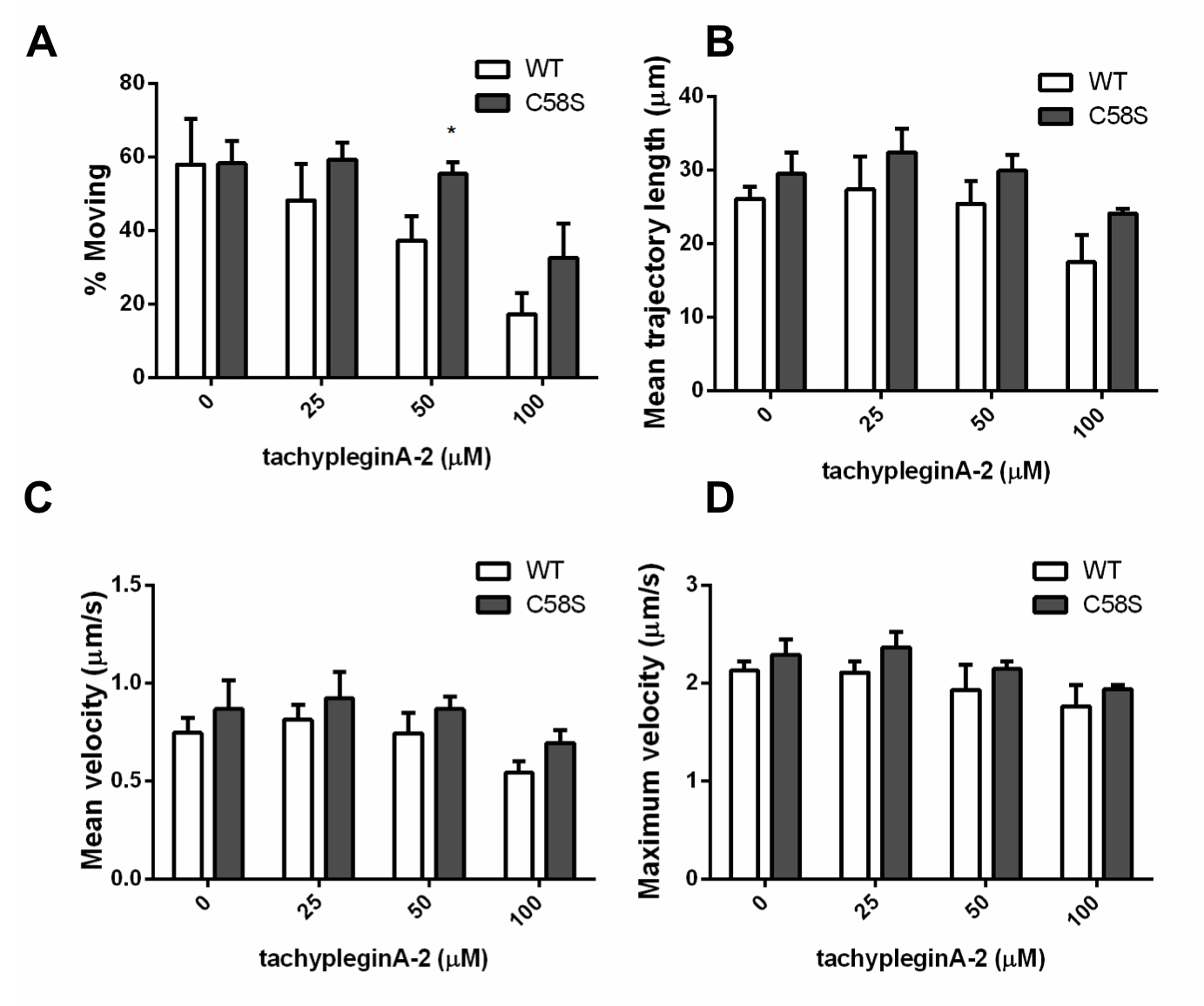

Supplement: Figure S6 — Non-normalized motility parameters of the knock-in parasites upon tachypleginA-2 treatment. Graphs comparing the (A) percent moving, (B) mean trajectory length, (C) mean velocity and (D) maximum velocity of WT (white bars) and C58S (grey bars) knock-in parasites in the 3D motility assay. The total number of WT parasites analyzed was 7,123 for DMSO, 4,662 for 25 µM tA-2, 5,255 for 50 µM tA-2 and 4,328 for 100 µM tA-2; the total number of C58S parasites analyzed was 5,484 for DMSO, 3,325 for 25 µM tA-2, 4,587 for 50 µM tA-2 and 4,417 for 100 µM tA-2. Data shown are the results of three independent experiments, with each experiment performed in triplicate. Datasets were compared by two-way ANOVA (* p < 0.05); error bars = standard deviation. (TIF) [file pone.0098056.s006.tif]
