# Supplementary material for: Identification of T. gondii Myosin Light Chain-1 as a Direct Target of TachypleginA-2, a Small-Molecule Inhibitor of Parasite Motility and Invasion
Source: PLoS One. 2014 Jun 3;9(6):e98056. doi: 10.1371/journal.pone.0098056 (PMC4043638; doi:10.1371/journal.pone.0098056)
Supplement: Table S1 — Primers used in this study. (DOCX) [file pone.0098056.s008.docx]

| Primer | Name | Sequence (5’ 🡪 3’); restriction sites are underlined |
| --- | --- | --- |
| 1 | *EcoR*I-FLAG-TgMLC1-Fwd | GAATTCATGGATTACAAGGATGACGACGATAAGATGAGC |
| 2 | TgMLC1-*Bgl*II-Rev | AGATCTTTACTCCCTTCGCTCGAGCATTGC |
| 3 | TgMLC1S57AFwd | TGTGAGTCGCCCGCTTGCCGCGAGG |
| 4 | TgMLC1S57ARev | CCTCGCGGCAAGCGGGCGACTCACA |
| 5 | TgMLC1C58S2Fwd | TGAGTCGCCCTCTTCCCGCGAGGG |
| 6 | TgMLC1C58S2Rev | CCCTCGCGGGAAGAGGGCGACTCA |
| 7 | TgMLC15’flank*Kpn*IFwd | ACGAGGTACCAGTGAATGAAGCAGGC |
| 8 | KozakATGFLAGTgMLC1Rev | TCGTCGTCATCCTTGTAATCCATCTTGAGACCAGTTGGCAGGCGC |
| 9 | KozakATGFLAGTgMLC1Fwd | GCGCCTGCCAACTGGTCTCAAGATGGATTACAAGGATGACGACGA |
| 10 | TgMLC1DHFR3’UTRRev | ACGGGCAGCTTCTGTGGGCTGCATTACTCCCTTCGCTCGAGCATT |
| 11 | TgMLC1DHFR3’UTRFwd | AATGCTCGAGCGAAGGGAGTAATGCAGCCCACAGAAGCTGCCCGT |
| 12 | DHFR3’UTR*Hind*III | ATATTAAAGCTTGCGGTGTCACTGTAGCCTGCCAG |
| 13 | TgMLC13’flank*Bam*HIFwd | ATTAGGATCCGTTCTAGGCGCGAG |
| 14 | TgMLC13’flank*Xba*IRev | GTCCTCTAGACGATACATTTGTG |
| 15 | GRA1*Kpn*IFwd | TCTGGATGGTACCGGTGCTCGTATGCGACACG |
| 16 | SAG3’UTR*Bgl*II*Kpn*IRev | CTGGGTACCAGATCTGGGGCAAGAATTGTGTTAACC |
| 17 | TgMLC15’flankupstrFwd | AATGTCCGTAGCAGGCAGCA |
| 18 | GRA1*Bgl*IIRev | GCTAGCCGAGATCTCTTGCTTGATTTCTTCAAAG |
| 19 | TgMLC1+1587(Exon3Start)Fwd | CACAAGTGACCAGATCGACTACAG |
| 20 | TgMLC13’flankdownstr+829Rev | CCTTTCAAGTCCGTTCGCAACCT |
