# Supplementary material for: Identification of T. gondii Myosin Light Chain-1 as a Direct Target of TachypleginA-2, a Small-Molecule Inhibitor of Parasite Motility and Invasion
Source: PLoS One. 2014 Jun 3;9(6):e98056. doi: 10.1371/journal.pone.0098056 (PMC4043638; doi:10.1371/journal.pone.0098056)
Supplement: Supporting Information S1 — (DOC) [file pone.0098056.s009.doc]

**Supporting Information**

**Experimental**

*General*

Thin layer chromatography (TLC) analysis was performed using glass plates coated with silica gel (with fluorescent indicator UV_254_). Developed plates were air dried and analysed under a UV lamp (254/365 nm). Flash chromatography was performed using silica gel (40-63 µm, Fluorochem). Low resolution (LR) and high resolution (HR) electrospray mass spectral (ES-MS) analyses were acquired by electrospray ionisation (ESI), electron impact (EI) or chemical ionisation (CI). These were acquired within the School of Chemistry, University of St Andrews. Nuclear magnetic resonance (NMR) spectra were acquired at room temperature on either a Bruker Avance 300 (^1^H, 300.1 MHz; ^13^C, 75.5 MHz), a Bruker Avance II 400 (^1^H, 400.1 MHz; ^13^C, 100.6 MHz), a Bruker Avance 500 (^1^H, 500 MHz; ^13^C, 125.7 MHz) or a Bruker Avance III 500 (^1^H, 500.1 MHz, ^13^C, 125.7 MHz) spectrometer and in the deuterated solvent stated. All NMR spectra were acquired using the deuterated solvent as the lock. Coupling constants (*J*) are quoted in Hz and are recorded to the nearest 0.1 Hz. The following abbreviations are used; s, singlet; d, doublet; t, triplet; m, multiplet and br, broad. Chemical shifts are expressed as δ in units of ppm. ^13^C NMR spectra were recorded under the same conditions and solvents using the PENDANT sequence mode. Data processing was carried out using the TOPSPIN 2 NMR program (Bruker UK Ltd).

#### Synthesis of (3E,5E)-3,5-dibenzylidene-1-propylpiperidin-4-one, tachypleginA-2

Benzaldehyde (1.51 mL, 14.87 mmol, 2.1 eq.) was added to a suspension of *N*-^n^propyl-4-piperidone (1.00 g, 7.08 mmol) in a solution of acetic acid saturated with dry HCl gas (5.0 mL). The reaction was stirred at room temperature for 24 h before the addition of a saturated aqueous solution of K_2_CO_3_ (10.0 mL) and acetone (5.0 mL). The resulting mixture was then stirred for 30 min and the titled compound, **tachypleginA-2** collected and recrystallised from 95% ethanol as a yellow needle crystalline solid (1.57 g, 70%) **m.p.**: 114.0-114.5 °C; **^1^H NMR** (300 MHz, CDCl_3_): δ 0.80 (t, *J* 7.30 Hz, 3H), 1.47 (h, *J* 7.3 Hz, 2H), 2.54-2.62 (m, 2H), 3.89 (s. 4H), 7.63-7.48 (m, 10H), 7.72 (s, 2H); **^13^C NMR** (75 MHz, CDCl_3_): δ 12.2, 20.8, 55.2, 59.7, 129.0, 129.4, 130.8, 133.8, 135.7, 136.8, 187.9; **HRMS** (ESI) calculated for C_22_H_24_NO: 318.1858, found: 318.1851.

**Synthesis of (*3E,5E*)-3,5-bis(4-(prop-2-ynyloxy)benzylidene)-1-propylpiperidin-4-one, tachypleginA-4**

4-(Prop-2-ynyloxy)benzaldehyde (2.38 g, 14.87 mmol, 2.1 eq.) was added to a suspension of *N*-*^n^*propyl-4-piperidone (1.00 g, 7.08 mmol) in a solution of acetic acid saturated with dry HCl gas (5.0 mL). The reaction was stirred at room temperature for 24 h before the addition of a saturated aqueous solution of K_2_CO_3_ (10.0 mL) and acetone (5.0 mL). The resulting mixture was then stirred for 30 min and the titled compound, **tachypleginA-4** collected and recrystallised from 95% ethanol as a yellow needle crystalline solid (2.35 g, 78%). **m.p.** 189.8-190.8 °C; **^1^H NMR** (300 MHz, CDCl_3_): δ 0.95 (t, *J* 7.31 Hz, 3H), ), 1.40-1.61 (m, 2H), 2.55 – 2.71 (m, 4H) , 4.30 (s, 4H), 4.71 (d, *J* 2.28 Hz, 4H) 6.90-6.96 (d, *J* 9.12 Hz, 4H), 7.30-7.36 (d, *J* 9.12 Hz, 4H), 7.82 (s, 2H). **^13^C NMR** (75 MHz, CDCl_3_): δ 11.8, 20.5, 54.9, 55.8, 59.3, 75.9, 78.2, 115.0, 128.9, 131.9, 132.2, 135.8, 158.1, 187.4; **HRMS** (ESI) calculated for C_28_H_28_NO_3_: 426.2042; found 426.2054.

**Inseparable diastereomeric mixture of diethyl 2,2'-(((4-oxo-1-propylpiperidine-3,5-diyl)bis((4-(prop-2-yn-1-yloxy)phenyl) methylene))bis(sulfanediyl))diacetate S1**

Et_3_N (0.13 mL, 0.951 mmol, 2.10 eq.) was added to a solution of **tachypleginA-4** (150 mg, 0.474 mmol) in DCM (5 mL) followed by the dropwise addition, at 0°C, of a solution of ethyl-2-mercaptoacetate (0.04 mL, 0.951 mmol, 2.10 eq.) in DCM (1 mL). The reaction mixture was then stirred at room temperature for 24 hours before being concentrated *in vacuo* to give a yellow oil. Purification by column chromatography (hexanes/EtOAc: 7/3) afforded **S1** as a colourless oil as a mixture of at least 3 diastereoisomers (**D1**, **D2** and **D3,** see Figure S2B for possible structures) as identified by NMR spectroscopy (181 mg, 60 %). **^1^H NMR** (300 MHz, CDCl_3_): δ 0.60 – 0.95 (m, 3H), 0.99 - 1.19 (m, 3H), 1.23-1.36 (m, 2H), 2.49 – 2.51 (m, 2H), 2.65 – 3.02 (m, 8H), 3,.35 – 3.49 (m, 2H) 3.73 - 4.10 (m, 4H), 4.39 – 4.42 (m, 0.1H, Ar-CH**_D1_**-S-); 4.50 – 4.54 (m, 0.62H, Ar-CH**_D1_**-S- and Ar-CH**_D2_**-S-), 4.67 – 4.70 (m, 4H), 4.71 – 4.78 (m, 1.29H, Ar-CH**_D3_**-S-), 7.02 – 7.39 (m, 8H); **^13^C NMR** (75 MHz, CDCl_3_): δ 11.6, 13.9, 20.4, 32.8, 33.3, 34.4, 34.7, 47.5 (Ar-CH**_D3_**-S-), 48.7 (Ar-CH**_D1_**-S- and Ar-CH**_D2_**-S-), 52.1, 52.7, 53.5, 54.2, 54.5, 55.6, 58.9, 60.0, 61.3, 128.9, 130.5, 133.3, 136.2, 169.9, 170.3, 187.5; **HRMS** (ESI) calculated for C_36_H_44_NO_7_S_2_: 666.2559; found 666.2563.

**Synthesis of (3*E*,5*E*)-3,5-di-d5-benzylidene-1-propylpiperidin-4-one, D10-tachypleginA-2**

Benzaldehyde-*d*_5_ (0.21 mL, 2.087 mmol, 2.1 eq.) was added to a suspension of *N*-*^n^*propyl-4-piperidone (0.15 mL, 0.994 mmol) in a solution of acetic acid saturated with dry HCl gas (5.0 mL). The reaction was stirred at room temperature for 24 h before the addition of a saturated aqueous solution of K_2_CO_3_ (10.0 mL) and acetone (5.0 mL). The resulting mixture was then stirred for 30 min and the titled compound, **D10-tachypleginA-2** collected and recrystallised from 95% ethanol as a yellow needle crystalline solid (235 mg, 72%). **m.p.** 202.7-203.2 °C; **^1^H NMR** (300 MHz, CDCl_3_): δ 0.87 (t, *J* 7.9Hz, 3H), 1.49 (h, *J* 7.3 Hz, 3H), 2.52-2.57 (m, 2H), 3.88 (s, 4H), 7.86 (s, 2H); **^13^C NMR** (126 MHz, CDCl_3_): δ 11.7, 20.3, 54.5, 58.9, 128.1 (t, *J* 23.51 Hz), 130.0 (t, *J* 23.51), 132.7, 135.0, 136.9, 187.1; **HRMS** (ESI) calculated for C_22_H_14_D_10_NO: 328.2485; found 328.2496.
